# Preliminary experimental support for a vulnerability theory of emotional bonding

**DOI:** 10.1038/s41598-025-24119-z

**Published:** 2025-11-17

**Authors:** Robert Epstein, Amanda Newland, Camille Reid

**Affiliations:** 1 American Institute for Behavioral Research and Technology, 1035 E Vista Way #120, Vista, CA 92084 USA; 2 University of the South Pacific, Suva, Fiji

**Keywords:** Vulnerability, Emotional bonding, Intimate relationships, Vulnerability theory of emotional bonding, VTEB, Health care, Psychology, Psychology

## Abstract

In an internet-based study with a diverse group of 1000 adult participants in the US, support was found for a “vulnerability theory of emotional bonding” (VTEB), according to which the strength of a new emotional bond can be predicted by measures of the vulnerability that each of two persons shows with respect to one another, where vulnerability is defined by measures of states of need and empathy. In a pre-manipulation-post-manipulation design, participants were first asked questions regarding their current states of need and empathy, then shown a photograph of a male confederate and asked questions regarding their feelings toward him. Participants were then randomly assigned to one of four groups in which they viewed a video showing the confederate responding to a set of questions about cancer but showing, in each of the four respective groups, reactions indicating either (1) low need and low empathy, (2) low need and high empathy, (3) high need and low empathy, or (4) high need and high empathy, following which participants were again asked questions regarding their feelings toward the confederate. As predicted, new bonds were weakest when need and empathy states were low, and highest when need and empathy states were high. The study suggests that further research may make it possible to develop a formal and predictive theory of emotional bonding of the form *B* = *f* (*N*_1_, *N*_2_, *E*_1_, *E*_2_), where *E* and *N* are measures of empathy and need, respectively, and *B* is the strength of the resulting bond.

**Supplementary Information:**

The online version contains supplementary material available at 10.1038/s41598-025-24119-z.

## Introduction

Although love, and even liking, are generally portrayed as mysterious phenomena in novels and movies, hundreds of studies in a specialty called “relationship science” suggest that the process of emotional bonding is actually both orderly and predictable. In the present paper, we will describe a quantitative theory of liking and loving we call the “vulnerability theory of emotional bonding” (VTEB), and we will present data from a randomized, controlled experiment that lend support to this theory.

Let’s think about this issue first from a common sense perspective and then by looking at some relevant peer-reviewed studies. When two people are in a dangerous situation—say, two soldiers are in a foxhole in the middle of an intense battle—they sometimes form a strong emotional bond that can last a lifetime^1^. For purposes of this paper, we will define an emotional bond as a strong and lasting connection between two individuals that is developed through shared intense emotions, understanding, and empathy^2,3^. This doesn’t just happen in the movies. In 2012, when a disturbed young man shot many innocent people (including 20 young children) at the Sandy Hook Elementary School in Newtown, Connecticut, relatives of people in the school waited nervously in a firehouse nearby to learn the fates of their loved ones. Two of those people, Carlee Soto and Erica Lafferty, got bad news. Both Carlee’s sister, a teacher at the school, and Erica’s mother, the school principal, were killed. The grief they shared bonded them closely and launched them on a nationwide effort to strengthen gun safety laws^4^.

In cases like these, both people feel vulnerable—that is, they feel like they are helpless or weak or in danger (we’ll talk about vulnerability more precisely later). In other situations, each member of a dyad might feel vulnerable in a different way. For example, when a woman (“Pat”) stops to help another woman (“Alex”) who has collapsed on the sidewalk, Pat might feel vulnerable because she has no idea how to help and perhaps because she lacks the physical strength to help Alex to her feet; whereas Alex might feel vulnerable because she is ill or has been injured. Again, it’s not hard to find real examples. In 1952, a woman named Dolores saved a young man from drowning in a lake in Spokane, Washington. That encounter led to a marriage that lasted 64 years, until Dolores passed away in 2018^5^. In most tellings of “The Little Mermaid” [e.g., ^6^], the mermaid saves the Prince from a ship wreck—in other words, from drowning—and later, when she washes ashore with no voice, the Prince rescues her. They meet under conditions of high vulnerability and, needless to say, they fall deeply in love and live happily ever after. The romances that develop in classic movies such as “Breakfast at Tiffany’s,” “Pretty Woman,” and “Titanic,” can all be analyzed from a vulnerability perspective.

What kinds of parameters might be important when vulnerability produces an emotional bond? One might try to measure the magnitude of the vulnerability in some way, and that magnitude might be different for each person in the dyad. Presumably, the lower the magnitude, the weaker the bond that might be generated. One might also find an imbalance, with one person feeling more vulnerable than the other. In the extreme case, one person might feel highly vulnerable and the other person not vulnerable at all; this case might be relevant to the common and painful scenario called “unrequited love”^7,8^.

Interactions in which one or both members of a dyad are vulnerable actually involve two different emotional states. Someone who has been injured or is in danger feels *need*, and the other party might or might not feel some degree of *empathy* in response to that need. Both need and empathy can presumably be measured^9–11^. As we progress, we will show how these ideas can be shaped into a formal and testable theory of emotional bonding. First, let’s look at the wide range of studies that measure various factors that affect emotional bonding.

## Relevant studies

Studies relevant to VTEB—in other words, relevant to the role that vulnerability plays in emotional bonding—have been conducted since at least the 1970s [e.g., ^12–15^]. Supplementary Table S1 breaks down such studies into 36 categories and shows relevant citations. In this section of our essay, we will focus on just two of those categories which we believe demonstrate the power of vulnerability especially well. For additional examples of multiple areas of emotional bonding research that can reasonably be interpreted from a vulnerability perspective, see Supplementary Text S1 and S2.

### Physical danger

Soldiers in combat settings have long been known to form strong emotional bonds, sometimes lasting a lifetime^16^. The best known experimental study demonstrating the power that danger has to create an emotional bond is often referred to simply as “the bridge study.” This field study made clever use of two bridges located over the Capilano River in North Vancouver, Canada. One bridge was a suspension bridge that swayed and wobbled 230 feet above the river; the second was a solid, stable wood bridge only 10 feet above the river. In one condition, an attractive female confederate stopped and spoke with men who were crossing either one bridge or the other (as long as they were not accompanied by any females). In the presence of the confederate, the men wrote brief stories in reaction to Thematic Apperception Test (TAT) pictures, and the confederate also gave the men her phone number in case they wanted to call with questions. The result was clear: “Sexual content of stories written by participants on the fear-arousing bridge and tendency of these participants to attempt post-experimental contact with the interviewer were both significantly greater”—that is, greater than they were on the stable bridge^14^. No such effect was found when the procedure was repeated with a male confederate and male participants.

More recent studies have found support for the vulnerability hypothesis in different ways. For example, a study by Bastian et al. reported that strangers sharing a pain experience (holding their hands in ice water) formed emotional bonds, whereas strangers holding their hands in room-temperature water did not^17^. In a more recent study of this sort, researchers reported that relationship satisfaction in newlywed couples increased—at least temporarily—when they experienced a natural disaster together—Hurricane Harvey in Harris County, Texas^18^. For more examples, see Table S1. Counselors and therapists trying to help failing relationships sometimes advise couples to put themselves in dangerous and exciting situations—say, by riding a roller coaster together—to create or strengthen an emotional bond^19–21^.

### Reciprocal self-disclosure

The famous bridge study mentioned above was coauthored by relationship expert Arthur Aron of the State University of New York at Stony Brook. Another classic study by Aron and his colleagues showed that emotional bonds could be strengthened when vulnerability is heightened simply by reciprocal self-disclosure^22^. The study demonstrated this effect both for strangers and for people already in a relationship. When you trade secrets with someone—a practice children sometimes engage in or a couple sometimes tries on a first date—you and your fellow confessor put yourselves at risk. Couldn’t your partner someday reveal your secret to others, subjecting you to shame or punishment? A recent study conducted in The Netherlands with 208 children and one of their parents (208 dyads in total) replicated the Aron et al. finding, demonstrating that children felt more love for their parent when they gave away secrets than when they engaged in innocent small talk^23^. Self-disclosure has also been found to be positively correlated with success in friendships, dating, and marriages^24^.

Self-disclosure was central to a theory of emotional bonding—the Interpersonal Process Model of Intimacy (IPM)—published as a book chapter by Reis and Shaver (1988). The theory was not quantitative, and it was not supported by empirical findings, but it described a process of emotional bonding almost identical to the one that is central to VTEB. According to IPM, self-disclosure was the mechanism by which need was expressed—or, more specifically, “personal desires, fantasies, anxieties, and emotions”^25^. A bond can begin to form if the listener is “responsive,” or, more specifically, if he or she “responds supportively and empathically” of their partner’s disclosure^25^. The bond is formed when the vulnerable person “perceives the partner’s responsiveness”—that is, by interpreting the partner’s response to have “understanding, validation, and caring”^25^.

Let’s consider a few ways in which VTEB and IPM differ. We do so because looking at these differences will allow us to pinpoint some important aspects of VTEB. Note, first of all, that IPM is unidirectional, whereas VTEB is, depending on the situation, either unidirectional or bidirectional. When Pat, the passerby, stops to help Alex, the woman who has fallen, either or both women might feel both need and empathy. When the vulnerability is bidirectional—as it likely is, for example, for the two soldiers in a foxhole during a battle—we might expect the resulting emotional bond to be strongest.

Second, note that according to IPM, not only is responsiveness (or “empathy”) important; so is the *perception* of empathy by the person in need. VTEB omits this element of the interaction. That perception might certainly move things along, but we doubt that it is necessary. When the little mermaid saves the unconscious Prince from drowning, he does not perceive her empathy. He learns about the rescue much later, long after he has already bonded with her for other reasons. The need and empathy are present in the situation, however, and they certainly cause the little mermaid to begin to bond with the Prince. The process by which bonding occurs is often complex; we’ll say more about this issue below, as well as in our Discussion.

Note also that IPM is applied only to self-disclosure. The reach of VTEB is much greater. It easily applies to most of the 36 of the scenarios in Table S1, for example—to *any* situation in which either a unidirectional or bidirectional need/empathy pairing might exist.

As we will explain below, VTEB also lends itself to quantification, which means it could at some point be used to make predictions. In our view, theories in the behavioral and social sciences are all too often simply descriptive rather than mathematical [cf. ^25–28^]. Moreover, they often borrow categorical terms from the vernacular (e.g., eye colors, racial designations, gender categories, or sexual orientation labels), failing to recognize that categorical variables are rare in nature^29^. In multiple sciences—among them, gender studies, palaeontology, population genetics, sociology, and quantum mechanics—substantial progress was made only after scientists acknowledged that key variables are continuous, not categorical^29–34^. One advantage of doing so is that continuous variables lend themselves to quantification and prediction. We will say more about these issues when we describe VTEB in more detail below.

## Vulnerability, need and empathy

**Fig. 1:**
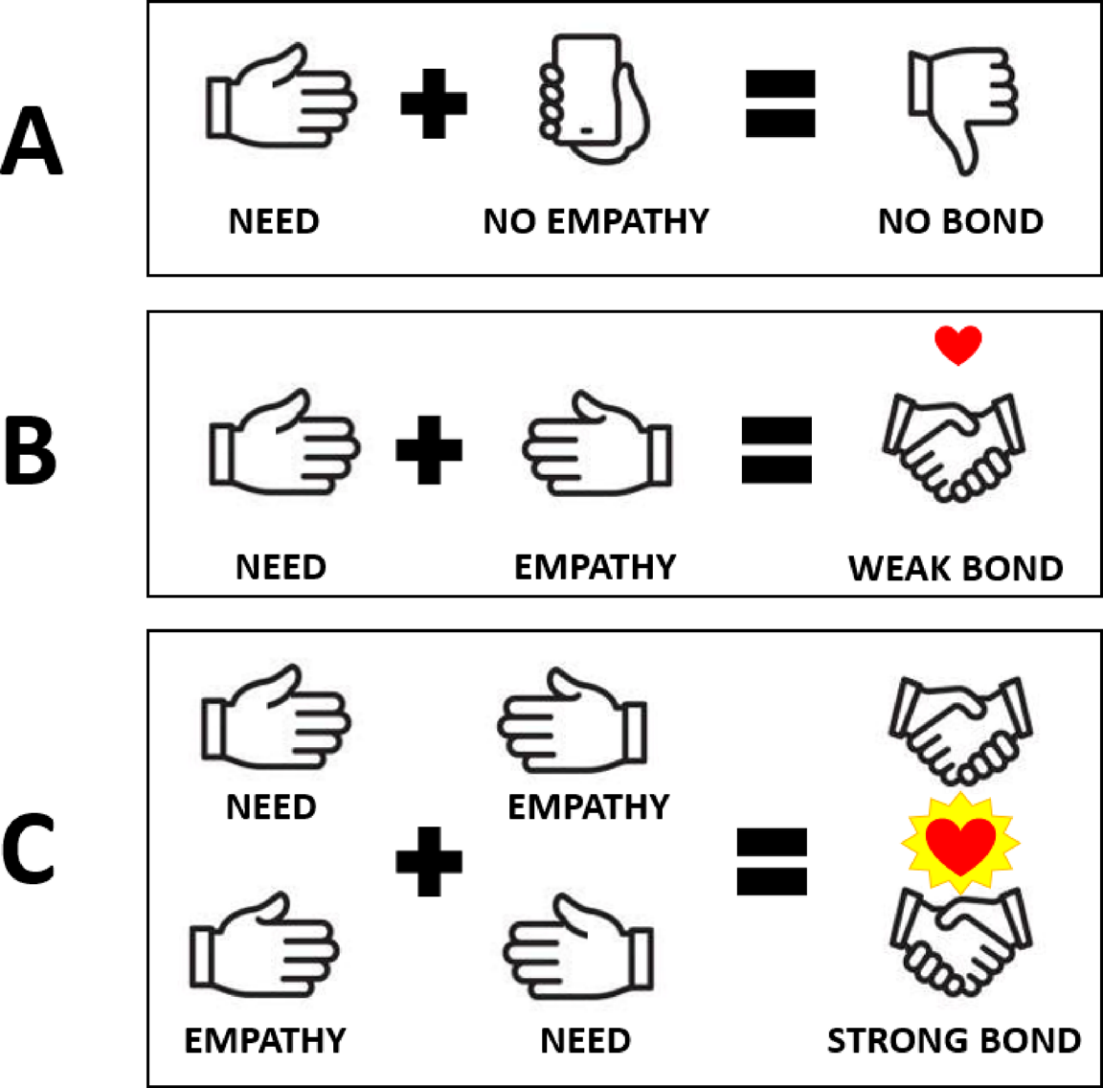
The role that need and empathy play in emotional bonding. (**A**) No bond is formed when one person expresses a need, and the other person fails to empathize. (**B**) A weak bond is formed when one person expresses a need, and the other person expresses empathy. (**C**) A strong bond is formed when two people in a dyad express need, and each person responds with empathy.

We find it useful to identify two complementary components of vulnerability: need and empathy. A roller-coaster scenario demonstrates how these two components might be expressed. If two people—Alex and Pat—share the same car on a frightening roller coaster, there are three ways in which need and empathy might be expressed: (1) Only one of these individuals (say, Alex) might be terrified (high need), and Pat might stay calm (low need) and might also show no empathy (low empathy) toward Alex. An emotional bond is unlikely to occur in this scenario (Fig. 1A). (2) Alex might be terrified (high need), and Pat, although calm (low need), might try to comfort Alex (high empathy). In this scenario, a week emotional bond might be formed (Fig. 1B), and, of course, the same pattern might occur in the other direction: Pat might be needy, and Alex empathetic. (3) Both Pat and Alex might be both needy and empathetic, in which case a strong emotional bond might be formed, at least temporarily (Fig. 1C). One interesting aspect of these bonding scenarios is that bonding might occur *even if the two people are strangers*^35–37^.

The need that either person in the dyad expresses could be an emotional need, physical need, or a combination of both. Examples of physical needs that past research has shown to be important factors in mate selection include the need for physical protection^38–41^, or needs that could be provided for with material wealth such as food and shelter^42–44^. Emotional needs that might be relevant to VTEB fall under the rubric commonly referred to as “negative affect”^45^. This includes most negative emotions, such as sadness, fear, and anger^45^. The expression of a negative emotion can act as a signal to the other person in the dyad that the first person is needy. The second person then might express empathy by providing assistance or comfort.

Many researchers consider empathy to be a multidimensional construct, encompassing aspects such as cognitive empathy—the ability to correctly identify what another person is feeling—and emotional empathy—the ability to adopt or share the feelings someone else is experiencing^46–48^. Additionally, the researchers that developed the Interpersonal Reactivity Index (IRI) identified four different aspects of empathy: (1) Perspective taking—one’s tendency to adopt the point of view of others in everyday life, (2) Empathetic concern—one’s tendency to experience feelings of sympathy and compassion for others in distress, (3) Personal distress—one’s tendency to feel distress in response to another individual in distress, and (4) Fantasy—one’s tendency to imagine themselves in fictional situations^49^. It is our view that not all of these aspects are crucial when it comes to emotional bonding. Empathetic concern might be the closest previously developed definition we have found, but even that is not complete, because it does not delineate the expression of that empathetic concern.

Some studies characterize the expression of empathy as “responsiveness”^50–52^. For example, in Study 2 in a paper by Welker et al. with 62 couples, “romantic partner responsiveness” proved to be a key predictor of the extent to which what the authors labeled “passionate love” emerged. Welker and colleagues based their description of responsiveness on the Interpersonal Process Model of Intimacy (IPM)^25^, and defined it as “the extent to which people empathically validate others’ thoughts and feelings”^51^.

A more recent study conducted online in Dutch with more than 10,000 participants also found a strong association between “perceived partner responsiveness” and sexual attraction^50^. van Lankveld et al. adopted the concept of perceived partner responsiveness from Reis, defining it as “the extent to which one experiences the partner as being responsive to one’s emotional needs”^50^. In a recent review of studies that examined the role that vulnerability plays in “building authentic relationships”^53^, the authors concluded, “When one partner expresses openly and the other partner shows presence by positive engagement, it builds trust, intimacy… and a sense of belongingness as it results in the feeling of being heard, loved, supported, understood, and validated”^53^. What we are labelling empathy is captured in the authors’ phrase “shows presence by positive engagement.” Notably, this review also concludes that there is currently “a dearth of detailed research in understanding the nature of vulnerability”^53^.

In the present paper, consistent with earlier researchers, we will define “need” as *a state of mind in which one desires help or support from another person*, “empathy” as *a state of mind in which one reacts to another person’s need with a desire to provide help or support*, and “vulnerability” as *a state of mind in which one feels (a) an emotional or physical need*,* (b) empathy in response to the needs of another person*,* or (c) both need and empathy in a reciprocal interaction with another person* [cf. ^25,54,55^].

### Some theories of love

Social and behavioral scientists have proposed a number of theories of intimacy over the years. Perhaps the best known of these is Robert Sternberg’s “triangular theory of love,” according to which love has three key components—intimacy, passion, and commitment—that combine to make eight different kinds of love^28^. Sternberg later developed a love scale based on the theory. When attempting to validate the scale, Sternberg concluded that the data were “generally, but not completely supportive of the utility of the triangular love scale”^56^. Although, in our view, neither the triangular theory nor the related love scale have ever been strongly validated, the theory and scale are widely employed in both theoretical papers and empirical studies [e.g., ^57–62^]. Note that Sternberg’s theory labels possible components and types of love; it does not explain *why* love emerges in relationships.

Other major theories of love include the theory of passionate and companionate love^63^, the theory of love styles^64,65^, and the more recent quadruple theory^60^. Like Sternberg’s theory, they generally describe characteristics of loving relationships rather than explaining how or why love appears. Also relevant here is attachment theory, proposed in the 1960s by British psychiatrist John Bowlby. Although he focused on mother infant bonding, rather than on adult love, other researchers have since applied his theory to some aspects of adult love, especially to “attachment styles”^66^. Note that Bowlby’s fundamental idea has a strong vulnerability component: An infant, when in distress (weak, needy), cries out, and a caring mother then holds and soothes the infant (empathy), often satisfying the specific need (for example, by feeding the infant) and thus deactivating the “attachment system” [^67^, cf. ^69,69^].

Another interesting variant on the role that vulnerability plays in bonding is implied in a behavioral theory of intimacy proposed by behavior analysts James V. Cordova and Rogina L. Scott [^26^, cf. ^70^]. Like VTEB, their theory identifies vulnerability as the essential mechanism in producing emotional bonds, although they are talking about “vulnerable behavior” rather than vulnerable people. Specifically, they propose that:

intimacy is a process that emerges from a sequence of events in which behavior vulnerable to interpersonal punishment is reinforced by the response of another person. These intimate events result in an increase in the probability of behavior vulnerable to interpersonal punishment in the presence of the reinforcing partner. The process results in intimate partnership formation and reports of feeling intimate^26^.

“Behavior vulnerable to interpersonal punishment” brings us back to self-disclosure. When you admit to someone that you hear voices, that you were molested when you were a child, or that you once suffered a terrible embarrassment, you are, according to the authors, taking a great risk. The listener might ridicule you (that’s an example of punishment, in their view) or even shun you (another kind of punishment). If, instead, the listener “reinforces” the vulnerable behavior—perhaps by showing signs of sympathy (behavior we have identified as empathetic or responsive), intimacy might occur. Unfortunately, the authors, adhering to strict behavior analytic language, merely predict the definitional outcome of reinforcement, namely, that the vulnerable behavior becomes more likely—specifically, that there is “an increase in the probability of behavior vulnerable to interpersonal punishment in the presence of the reinforcing partner.” Where, one might ask, is the intimacy here? The authors refer to the *events* as intimate; in other words, they identify both the expression of vulnerable behavior and the reinforcement of such behavior as intimate. The interaction, they say, “results in intimate partnership formation and reports of feeling intimate.”

The authors don’t attempt to quantify the theory, and they offer no supporting data. We might also dismiss the theory as somewhat simplistic—an attempt to use behavior-analytic language to connect with theories like IPM. Rather than dismiss the theory, however, we take interest in the fact that the authors have added a feature to the concept of vulnerability that might apply to many different situations; namely, that we are vulnerable when we are at risk for being harmed. This applies not only to sharing secrets with someone but also to Alex when she has collapsed on the sidewalk, to the Prince when he is drowning, and to both soldiers in the foxhole—all situations in which strong emotional bonds might be formed.

## Vulnerability theory of emotional bonding (VTEB)

The specific theory we will test in the present paper—the “vulnerability theory of emotional bonding” (VTEB) was first described in an empirical study in a peer-reviewed journal in 2013 [^54^, cf. ^21^]. According to the theory, emotional bonds are created and strengthened when one person’s need is paired with another person’s empathy; as noted above, this can happen in one direction, or it can happen more-or-less simultaneously when two people empathize with each other’s needs. The theory can be stated formally as follows:

$$\:B=\:f({N}_{1},{N}_{2,}{E}_{1},\:{E}_{2})\:$$

where *N*_*1*_ and *N*_*2*_ are measures of the strength of the needs of two people who interact with each other, *E*_*1*_ and *E*_*2*_ are measures of the strength of the empathy those people express toward each other, and *B* is the strength of the emotional bond formed by the interaction. The theory is depicted visually in Fig. 1.

### The present study

In the present study we describe a randomized, controlled experiment in which we test some aspects of VTEB by varying levels of need and empathy expressed by a confederate. We predict, among other things, that where low levels of need and empathy expressed by our confederate match low levels of need and empathy in a participant, emotional bonding will be weak, and that where high levels of need and empathy expressed by our confederate match high levels of need and empathy in a participant, emotional bonding will be strong. We also suggest a way to summarize our results mathematically—a step, we hope, toward beginning to understand emotional bonding in a quantitatively rigorous way.

## Methods

### Participants

Participants were recruited on Amazon Mechanical Turk (MTurk) between June 3rd and July 5th, 2014. This was before concerns had been raised about the growing number of bots in that online subject pool [^71^, cf. ^72,73^]. Participants were required to be 18 or older and to be residents of the US. Participants were also required to have access to high-speed internet to stream a video in real time, as well as a functioning audio device on their computer.

Before cleaning we had data from 1146 participants. Participants were removed who reported an age lower than 18 or reported living outside the US. We also removed duplicate cases and incomplete cases. Participants were asked to report their English fluency on a Likert scale ranging from 1 to 10, where 1 was labeled “Not fluent” and 10 was labeled “Highly fluent.” Participants also reported their vision and hearing capabilities on a Likert scale ranging from 1 to 10, where 1 was labeled “Very poor” and 10 was labeled “Very good.” Participants were removed who reported an English fluency below 8, and vision or hearing below 5. After cleaning, we had data from 1121 people. Then, in order to equalize the size of the four groups we analyzed, data from the first 250 participants in each group were included in final analyses. Therefore, we analyzed data from a total of 1000 participants.

Our participants ranged in age from 18 to 76 (*M* = 34.1, median = 31, *SD* = 11.3). 54.3% (*n* = 543) of the participants identified themselves as male, 45.6% (*n =* 456) as female, and 0.1% (*n* = 1) as other. 90.4% (*n* = 904) of the participants identified themselves as heterosexual, 6.3% (*n* = 63) as bisexual, 2.2% (*n* = 22) as gay or lesbian, 0.6% (*n* = 6) as other, and 0.5% (*n* = 5) as unsure. 76.7% (*n* = 767) of the participants identified themselves as White, 8.0% (*n* = 80) as Black, 5.3% (*n* = 53) as Hispanic, 4.6% (*n* = 46) as Asian, 2.6% (*n* = 26) as multiracial, and 2.8% (*n* = 28) as other.

### Procedure

Participants were first shown a brief description of the study and then asked for their consent to proceed. They were then asked some basic demographic questions that were general enough to protect their identity. Then participants were asked seven questions about their current state of need, followed by seven questions about their current state of empathy. Each of the seven items on the state of need scale began with the phrase “I feel…” followed by a descriptor of need:

1. I feel tired.
2. I feel anxious.
3. I feel sad.
4. I feel hungry.
5. I feel thirsty.
6. I feel lonely.
7. I feel needy.

**Fig. 2:**
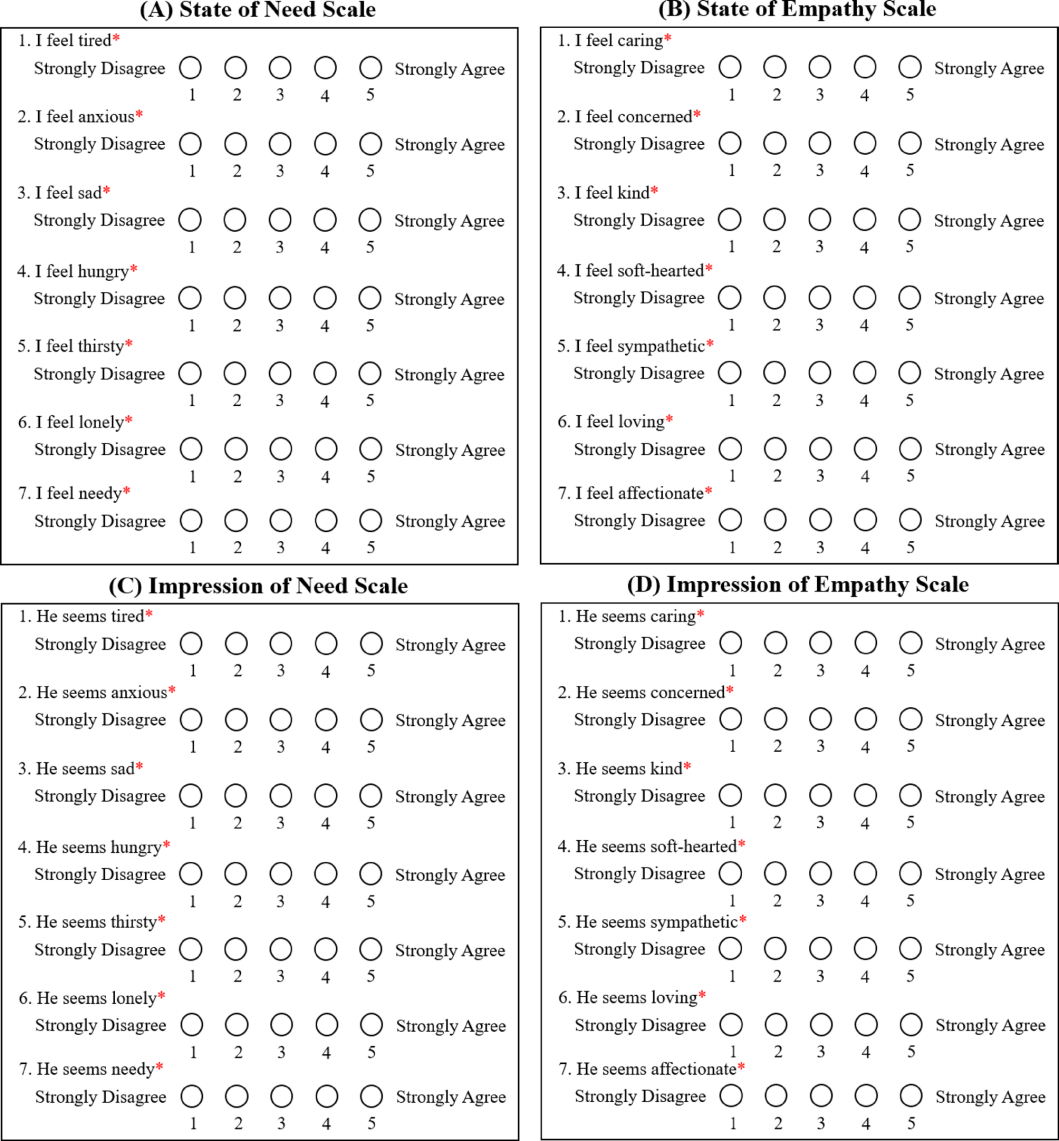
Need and empathy scales for the participants and the confederate. Participants rated their own states of need and empathy by answering the questions shown in boxes (**A**) and (**B**) above, respectively. Pre- and post-viewing of the video of the interview with the confederate, they rated the need and empathy of the confederate by answering the questions shown in boxes (**C**) and (**D**) above, respectively. The asterisk indicates “required.”

For each item, participants indicated their current state on a 5-point Likert scale, where 1 was labeled “Strongly Disagree” and 5 was labeled “Strongly Agree” (Fig. 2A). The seven items on the state of empathy scale followed the same structure; however, each statement ended with a descriptor of empathy, rather than need:

1. I feel caring.
2. I feel concerned.
3. I feel kind.
4. I feel soft-hearted.
5. I feel sympathetic.
6. I feel loving.
7. I feel affectionate.

Again, for each item, participants indicated their current state on a 5-point Likert scale, where 1 was labeled “Strongly Disagree” and 5 was labeled “Strongly Agree” (Fig. 2B).

**Fig. 3:**
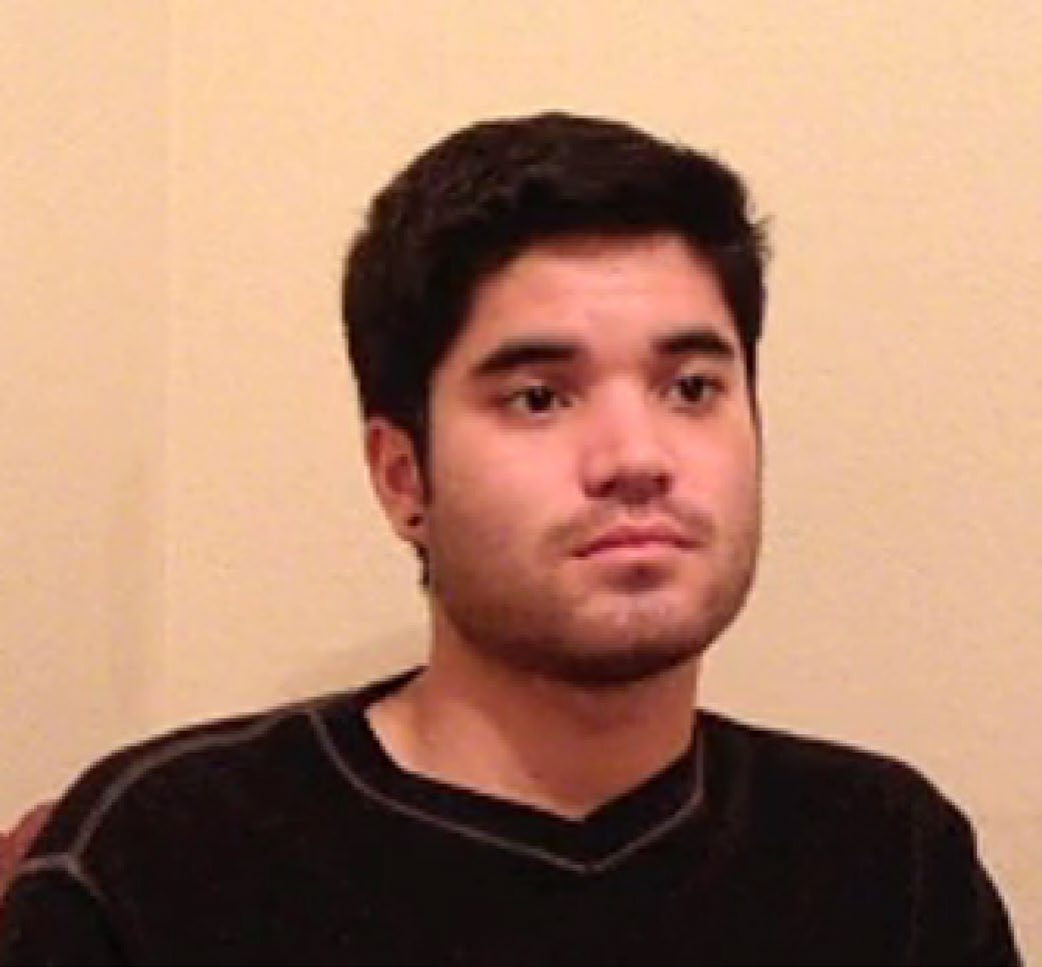
Picture of the confederate displaying neutral expression. Participants viewed a picture of the confederate displaying a neutral expression (shown above) while they completed their pre-manipulation ratings of their impression of the confederate’s state of need and empathy, as well as the emotional bond they felt toward the confederate.

Then participants were shown a picture of a confederate with a neutral facial expression (Fig. 3) and given the instruction: “Please look at this photo and answer the questions below.” Participants were then asked to rate their impression of the confederate’s state of need and empathy. The impression of need and impression of empathy scales used for this rating were identical to those used to assess the participants’ own states, with one exception: each item began with the phrase “He seems…” (Fig. 2C,D).

**Fig. 4:**
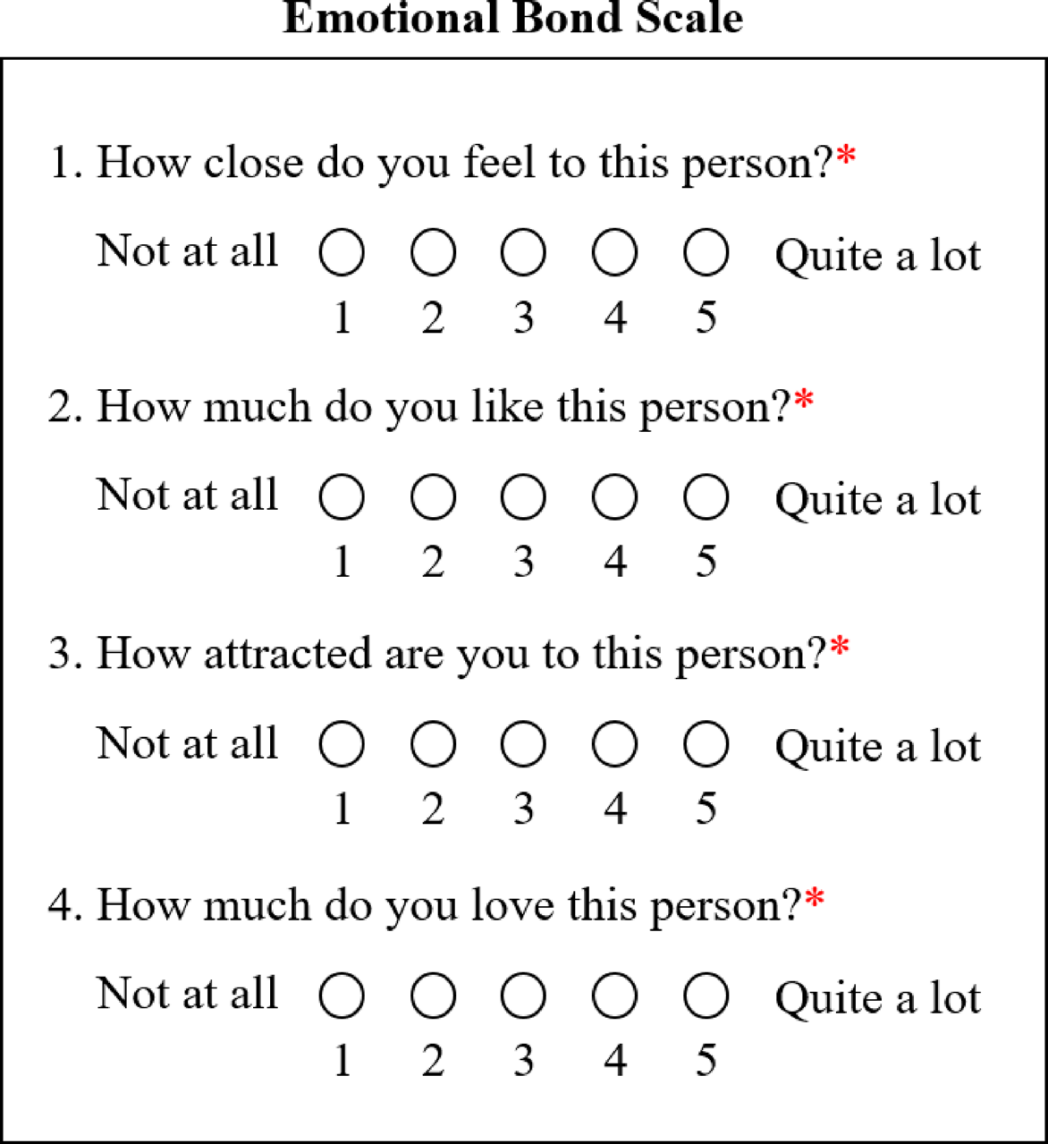
Emotional bond scale. Pre- and post-viewing of the video of the interview with the confederate, participants rated the level of emotional bond they felt toward the confederate by answering the questions shown in the box above.

Participants were then asked four questions about how emotionally bonded they felt toward the confederate (Fig. 4). Participants responded to each question on a 5-point Likert scale, where 1 was labeled “Not at all” and 5 was labeled “Quite a lot” (Fig. 4).

Participants were now given instructions about watching a video clip of a brief interview (the video was not present on the instructions page). They were told that the video was part of a series recorded to “promote cancer awareness.” Participants were instructed to watch the entire clip, ensure that their sound was on, and to turn up their volume. Upon completing their reading of the instructions, participants clicked on a “Continue” button to advance to the next screen. There participants watched a video of the confederate they had seen previously in a still photograph (Fig. 3). In the video, he answered questions about his experience with losing someone to cancer.

**Fig. 5:**
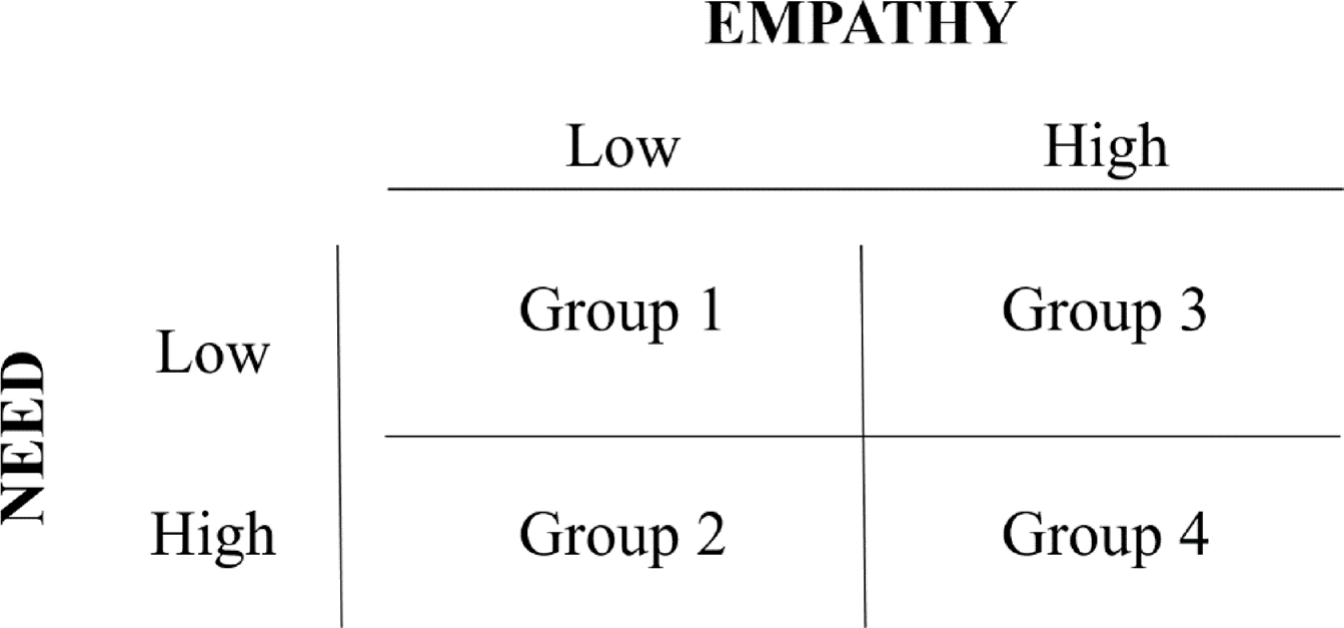
Levels of need and empathy expressed by the confederate in the videos shown to the four experimental groups. Participants were randomly assigned to one of four experimental groups, in which they saw varying levels of vulnerability displayed by the confederate during the video interview. In Group 1, the confederate expressed low levels of need and empathy (low vulnerability). In Group 2, the confederate expressed a high level of need and a low level of empathy (moderate vulnerability). In Group 3, the confederate expressed a low level of need and a high level of empathy (moderate vulnerability). In Group 4, the confederate expressed high levels of need and empathy (high vulnerability).

Without their knowledge, participants were randomly assigned to one of four experimental groups: (1) low need and low empathy, (2) high need and low empathy, (3) low need and high empathy, or (4) high need and high empathy (Fig. 5). For participants in Group 1 (low need–low empathy), the confederate in the video expressed very little sadness over the loss of his childhood neighbor who died from skin cancer and said he did not attempt to help comfort his family after his death. For participants in Group 2 (high need–low empathy), the confederate expressed extreme sadness over the loss of his cousin to leukemia and did not mention comforting others at all. For participants in Group 3 (low need–high empathy), the confederate discussed his time volunteering at a community center where he helped counsel and comfort cancer patients and their families. He did not mention any personal experiences with cancer. Lastly, for participants in Group 4 (high need–high empathy), the confederate expressed how devastated he and his family were by the recent loss of his mother to breast cancer. He also said that he had been attending conferences and community events to use his experience to help others and advocate for cancer research. The length of the video was controlled across the four groups, with each video lasting between 2m45s and 2m50s (*M* = 2m48s).

Following the video, the participants again completed the same fourteen questions about their current state of need and empathy, the same fourteen questions about their impression of the confederate’s state of need and empathy, and the same four questions about how emotionally bonded they felt to the confederate (Figs. 2 and 4). The study concluded with a message that thanked the participants for their involvement in the study and explained the general goals of the research, as well as how participants could withdraw their data from the study if they wished to do so.

### Statistical analysis plan

Because we employed a two-by-two factorial design, we began our analyses evaluating the pre- and post-manipulation scores on our four need and empathy scales (participants’ state of need, participants’ state of empathy, participants’ impression of the confederate’s state of need, and participants’ impression of the confederate’s state empathy) and our emotional bond scale. Specifically, when comparing the pre-manipulation or post-manipulation mean scores on the five scales between two groups, we used an independent *t*-test; with three or more groups, we used an analysis of variance (ANOVA). When comparing means between two groups, we also reported Cohen’s *d* as an estimate of effect size. When comparing means between three or more groups, we reported partial eta squared (η^2^) as an estimate of effect size. When comparing a pre-manipulation mean score to a post-manipulation mean score within the same experimental group, we used a paired samples *t*-test and reported the rank-biserial correlation coefficient (*r*) as an estimate of effect size.

We used a two-way ANOVA to test for main effects and an interaction between our two independent variables (level of need and level of empathy expressed by the confederate) and our dependent variable (pre-to-post-manipulation change in participants’ emotional bond to the confederate). We reported partial eta squared (η^2^) as an estimate of effect size for the two-way ANOVA.

Finally, we then used linear regression to determine if the participants’ states of need and empathy, and their impression of the confederate’s states of need and empathy, were significant predictors of the emotional bond the participants formed with the confederate. When reporting statistical significance, we adhered to the standards set forth in the 7th edition of the *Publication Manual of the American Psychological Association*^74^. Specifically, when the *p* value was greater than or equal to 0.001, we reported the actual *p* value; when the *p* value was below 0.001, we reported *p* < 0.001. Throughout our analyses, we used two-tailed tests.

## Results

### Pre- and post-manipulation measures

#### Pre-manipulation measures

Pre-manipulation (exposure to video), we found no statistically significant differences across the four experimental groups in the participants’ self-reported mean state of need scores (*M*_*Group1*_ = 14.46 [5.54], *M*_2_ = 14.42 [5.06], *M*_3_ = 14.01 [5.58], *M*_4_ = 13.74 [5.15], *F* = 1.03, *p* = 0.38 NS). Similarly, we found no statistically significant differences in the participants’ pre-manipulation mean state of empathy scores (*M*_*Group1*_ = 20.89 [7.09], *M*_2_ = 20.40 [7.32], *M*_3_ = 21.62 [7.53], *M*_4_ = 20.73 [7.30], *F* = 1.24, *p* = 0.29 NS).

For the participants’ ratings of their impression of the confederate’s state of need, pre-manipulation, we found no significant differences between the four groups in the mean scores (*M*_*Group1*_ = 16.38 [5.04], *M*_2_ = 16.42 [5.15], *M*_3_ = 16.76 [5.25], *M*_4_ = 16.36 [4.81], *F* = 0.34, *p* = 0.79 NS). In addition, for the participants’ ratings of their impression of the confederate’s state of empathy, we found no significant differences across the groups in the mean scores (*M*_*Group1*_ = 19.51 [6.00], *M*_2_ = 19.94 [5.78], *M*_3_ = 20.42 [5.38], *M*_4_ = 20.46 [5.99], *F* = 1.49, *p* = 0.22 NS).

Finally, for the participants’ ratings of the emotional bond they felt toward the confederate, pre-manipulation, we found no significant differences across groups in the participants’ mean ratings (*M*_*Group1*_ = 7.22 [2.24], *M*_2_ = 7.36 [2.39], *M*_3_ = 7.45 [2.48], *M*_4_ = 7.40 [2.45], *F* = 0.45, *p* = 0.72 NS).

### Post-manipulation measures

Post-manipulation, we found a significant difference in the mean scores of the participants’ impression of the confederate’s state of need across the four groups (*M*_*Group1*_ = 12.70 [4.47], *M*_2_ = 19.90 [4.62], *M*_3_ = 12.08 [4.20], *M*_4_ = 18.50 [4.34], *F* = 203.91, *p* < 0.001, η^2^ = 0.38). In the low-need groups (Groups 1 and 3), where the participants watched a video in which the confederate expressed a low level of need, there was a significant decrease in the participants’ pre- to post-manipulation ratings of the confederate’s state of need (Table 1). In the high-need groups (Groups 2 and 4), where the participants watched a video in which the confederate expressed a high level of need, there was a significant increase in the participants’ pre- to post-manipulation ratings of the confederate’s state of need (Table 1).

**Table 1:** Pre- and post-manipulation, participants’ impression of confederate’s state of need, mean scores across groups.

Level of need	Group	Pre-manipulation impression of need (*SD*)	Post-manipulation impression of need (*SD*)	Mean difference	*t*	*p*	Effect size (*r*)
Low-need	1	16.38 (5.04)	12.70 (4.47)	− 3.68	12.56	< 0.001	0.62
	3	16.76 (5.25)	12.08 (4.20)	− 4.68	16.53	< 0.001	0.72
High-need	2	16.42 (5.15)	19.90 (4.62)	3.48	− 13.05	< 0.001	0.64
	4	16.36 (4.81)	18.50 (4.34)	2.14	− 8.50	< 0.001	0.47

Post-manipulation, we also found a significant difference in the mean scores of the participants’ impression of the confederate’s state of empathy across the four groups (*M*_1_ = 17.36 [6.90], *M*_2_ = 26.37 [6.83], *M*_3_ = 29.45 [5.35], *M*_4_ = 28.79 [5.58], *F* = 202.18, *p* < 0.001, η^2^ = 0.38). In one of the low-empathy groups (Group 1), where the participants watched a video in which the confederate expressed a low level of empathy, there was a significant decrease in the participants’ pre- to post-manipulation ratings of the confederate’s state of empathy (Table 2). In the high-empathy groups (Groups 3 and 4), where the participants watched a video in which the confederate expressed a high level of empathy, there was a significant increase in the participants’ pre- to post-manipulation ratings of the confederate’s state of empathy (Table 2).

**Table 2:** Pre- and post-manipulation, participants’ impression of confederate’s state of empathy, mean scores across groups.

Level of empathy	Group	Pre-manipulation impression of empathy (*SD*)	Post-manipulation impression of empathy (*SD*)	Mean difference	*t*	*p*	Effect size (*r*)
Low-empathy	1	19.51 (6.00)	17.36 (6.90)	− 2.15	4.99	< 0.001	0.30
	2	19.94 (5.78)	26.37 (6.83)	6.43	− 15.85	< 0.001	0.71
High-empathy	3	20.42 (5.38)	29.45 (5.35)	9.03	− 23.08	< 0.001	0.83
	4	20.46 (5.99)	28.79 (5.58)	8.33	− 22.59	< 0.001	0.82

In the high-need groups (Groups 2 and 4), the confederate’s state of need was perceived to be substantially higher than in low-need groups (Groups 1 and 3) (*M*_*Low−Need*_ = 12.39 [4.34], *M*_*High−Need*_ = 19.20 [4.53], *t* = − 24.26, *p* < 0.001, *d* = − 1.54). In the high-empathy groups (Groups 3 and 4), the confederate’s state of empathy was perceived to be substantially higher than in low-empathy groups (Groups 1 and 2) (*M*_*Low−Empathy*_ = 21.87 [8.20], *M*_*High−Empathy*_ = 29.12 [5.47], *t* = − 16.45, *p* < 0.001, *d* = − 1.04).

**Fig. 6:**
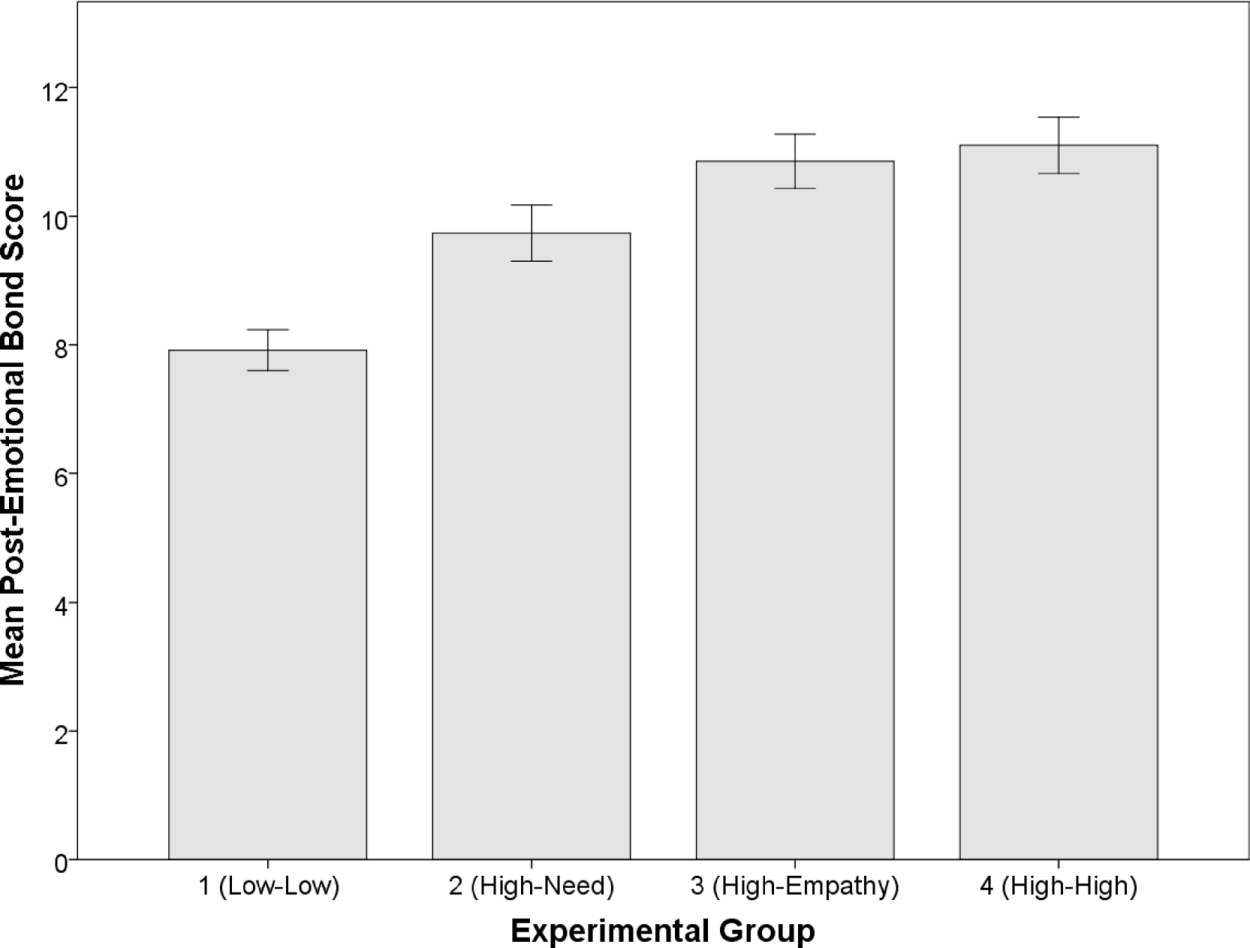
Increase in post-manipulation emotional bond scores reported by participants toward confederate across vulnerability conditions, groups 1 to 4. As the level of vulnerability expressed by the confederate increased, so did the emotional bond that the participants felt toward the confederate. Error bars were calculated with a 95% confidence interval.

Post-manipulation, we also found significant differences across the four groups in the mean ratings of the emotional bond the participants’ felt toward the confederate (*M*_*Group1*_ = 7.92 [2.55], *M*_2_ = 9.74 [3.49], *M*_3_ = 10.85 [3.39], *M*_4_ = 11.10 [3.50], *F* = 49.62, *p* < 0.001, η^2^ = 0.13). Comparisons of the pre- and post-manipulation emotional bond scores also showed significant increases in the emotional bond the participants felt toward the confederate in each experimental group (Table 3). As predicted, the magnitude of the pre- to post-manipulation shifts in emotional bond scores also increased as the level of vulnerability increased (Table 3). In other words, as the level of vulnerability that the confederate expressed in the interview increased, so did the strength of the emotional bond that the participants’ felt toward the confederate (Fig. 6).

**Table 3:** Pre- and post-manipulation, increase in mean emotional bond scores felt by participants toward confederate, groups 1 to 4.

Group	Pre-manipulation emotional bond score (*SD*)	Post-manipulation emotional bond score (*SD*)	Mean difference	*t*	*p*	Effect size (*r*)
1	7.22 (2.24)	7.92 (2.55)	0.70	− 4.55	< 0.001	0.28
2	7.36 (2.39)	9.74 (3.49)	2.38	− 13.22	< 0.001	0.64
3	7.45 (2.48)	10.85 (3.39)	3.40	− 20.64	< 0.001	0.79
4	7.40 (2.45)	11.10 (3.50)	3.70	− 21.10	< 0.001	0.80

### Main effects and interaction

**Fig. 7:**
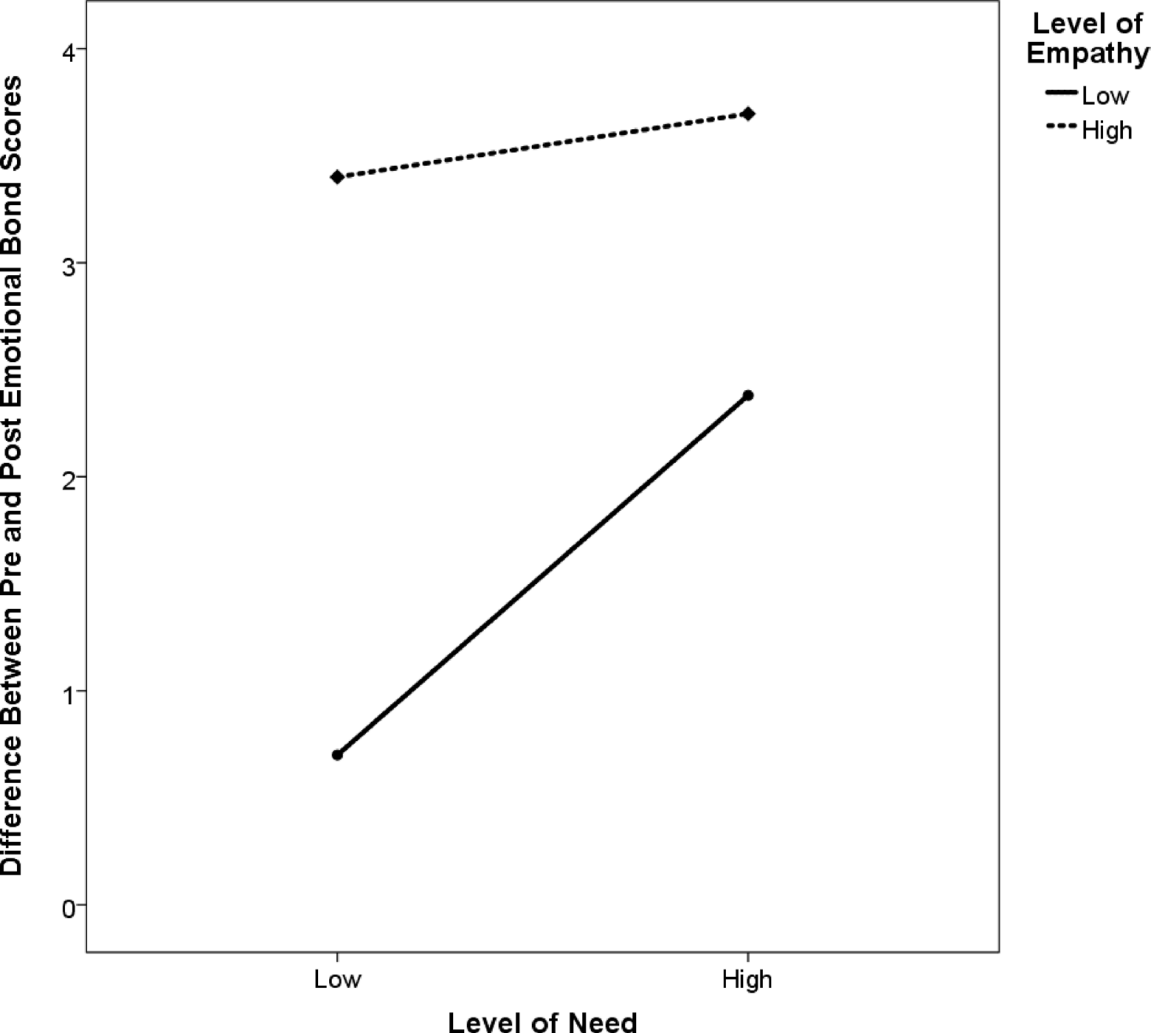
Difference between pre- and post-manipulation emotional bond scores by levels of need and empathy (on scale from − 16 to 16). A two-way factorial ANOVA found significant main effects for level of need and level of empathy, as well as a significant interaction between level of need and level of empathy.

We performed a two-way ANOVA to evaluate the effects of level of need and level of empathy displayed by the confederate on the pre-to-post increase in emotional bond scores reported by the participants. We found a significant main effect for level of need (*F*(1, 996) = 34.26, *p* < 0.001, η^2^ = 0.03); a significant main effect for level of empathy (*F*(1, 996) = 141.52, *p* < 0.001, η^2^ = 0.12); and a significant interaction between level of need and level of empathy (*F*(1, 996) = 16.81, *p* < 0.001, η^2^ = 0.02) (see Fig. 7).

### Predicting emotional bond from need and empathy

We conducted a linear regression to examine how predictive the participants’ ratings of their state of need, their state of empathy, their impression of the confederate’s state of need, and their impression of the confederate’s state of empathy were of participants’ ratings of the emotional bond they felt toward the confederate. The results showed that the participants’ state of need and empathy and their impression of the confederate’s state of need and empathy explained 47% of the total variability in the participants’ post-manipulation ratings of the emotional bond they felt toward the confederate. We found that the participants’ state of empathy, their impression of the confederate’s state of need, and their impression of the confederate’s state of empathy were significant predictors of emotional bonding (*F*(4, 995) = 222.23, *p* < 0.001, adj. *r*^2^ = 0.47) (Table 4).

**Table 4:** Participants’ state of need and empathy and impression of confederate’s state of need and empathy as predictors of emotional bond.

Variables	β	SE_B_	Standardized β	*p*
Intercept	0.77	0.36	–	–
State of need	0.03	0.02	0.05	0.07 NS
Impression of need	0.04	0.02	0.06	0.03
State of empathy	0.12	0.01	0.28	< 0.001
Impression of empathy	0.21	0.01	0.47	< 0.001

Given the original form of VTEB we stated in our Introduction,

$$\:B=\:f({N}_{1},{N}_{2,}{E}_{1},\:{E}_{2})\:$$

and defining our study participants to be Person 1 in the dyad and our confederate to be Person 2 in the dyad, we can use the participant’s self-reported states of need (N_1_) and empathy (E_1_) (post manipulation), as well as the participant’s impression of the confederate’s states of need (N_2_) and empathy (E_2_) (post manipulation), to populate the VTEB model as follows:

$$\:B=\left(.03*{N}_{1}\right)+\left(.04*{N}_{2}\right)+\left(.12*{E}_{1}\right)+\left(.21*{E}_{2}\right)+.77\:$$

It is presumably only the post-manipulation states of need and empathy that are important in predicting the magnitude of the emotional bond because these represent the peak or end of the interaction (say, when Pat has rescued Alex or when the bombs have stopped exploding).

## Discussion

### Major findings

This study presents a formal theory of emotional bonding that lends itself to empirical testing and quantification. According to the “vulnerability theory of emotional bonding” (VTEB), emotional bonds are created in dyads in situations in which one person’s needs—physical and/or emotional—are met by another person’s empathy. The bond can be unidirectional, as when Pat stops to assist Alex, or it can be bidirectional, as when Pat and Alex are companions in a foxhole. It is reasonable to believe that bidirectional emotional bonds will generally be stronger that unidirectional emotional bonds, but the present study does not test this assertion directly.

The magnitude of the emotional bond (*B*) that is formed is a function of the magnitude of the four variables mentioned above: *N*_1_, *N*_2_, *E*_1_, and *E*_2_. Although we presented a simple linear equation to summarize this relationship in the present study, we do not presume to know the correct or optimal form of this relationship. That said, it is notable that in the controlled experiment we described, our model accounted for 47% of the variability in *B*, the dependent variable.

Because we found main effects for both need and empathy, our data suggest that both factors can play a significant role in emotional bonding. The ordinal (non-crossing) interaction we found between need and empathy further suggests—at least in the present study—that the net impact of the two independent variables acting together is larger than what we would expect if each variable acted alone. That is potentially good news for two people riding on a roller coaster together on a first date.

### Limitations and future research

One potential limitation in our study is that our study design gives us no indication of how long an emotional bond will last after the initial interaction. It also does not allow us to assess how the bond might grow after repeated interactions. Past research suggests that repeated exposure can assist in the formation of a long-term emotional bond^75–78^.

Furthermore, while our results provide preliminary support for VTEB, not all of its predictions were fully supported. VTEB predicts that the strongest emotional bond will be formed when both need and empathy are present. While Group 4 (high-need and high-empathy) did have the largest pre- to post-manipulation increase in emotional bond scores out of our four experimental groups, the increase in scores in Group 4 was not substantially larger than the increase in Group 3 (low-need and high-empathy) (Table 3). This finding, along with the significant interaction we found between level of need and level of empathy (Fig. 7), suggests that empathy may play a stronger role in emotional bonding than need.

The main limitation we see in this study pertains to the variables over which we had control. Our data confirm that the four videos we showed to participants in Groups 1 to 4 differed from each other in the ways we had planned; that is to say, the video shown to members of Group 1 conveyed feelings of low need and low empathy; the second video conveyed feelings of high need and low empathy; and so on. In other words, we believe we did an adequate job of controlling two important independent variables: *N*_2_ and *E*_2_. We also showed that the emotional states portrayed in the videos had predictable effects on the emotional bonds of the participants. We did *not*, however, attempt to directly impact the emotional states of the participants before they viewed the videos.

Social psychologists and other researchers have long employed a variety of methods to manipulate the emotional states of their subjects before subjecting them to various manipulations. The classic study by Schachter and Singer (1962), for example, showed how different circumstances caused people to label their chemically-induced state of arousal in different ways^79^. Even the bridge study we mentioned earlier^14^ primed the level of vulnerability of its subjects before the experimental manipulation began. Recent studies continue to prime subjects in various ways before exposing them to different experimental conditions [e.g., ^80–84^]. A more thorough test of VTEB than the one we have presented would prime participants so they were experiencing different known levels of need and empathy before they were exposed to other people experiencing different levels of need and empathy.

These kinds of interactions could be studied with confederates in video recordings, as we chose to do, but they could also be studied with live confederates, or even with subjects who have been primed to be in the four different states of need and empathy we have discussed in this paper: low–low, low–high, high–low, and high–high. Experiments of this sort would have to be prepared and executed carefully, but they should be possible to conduct.

We believe that a number of studies of the sort we listed in Table S1 could be interpreted, or perhaps reinterpreted, from the VTEB perspective, but we can also envision investigations that could be conducted not only to test the theory but also to quantify it more precisely, thus allowing researchers to make increasingly accurate predictions about the emotional outcomes of various social interactions. Here are brief outlines of five types of procedures that we believe could shed further light on VTEB, and, in so doing, that could give us a more rigorous and, perhaps, more predictive understanding of emotional bonding:

1. In order to prime participants’ levels of need, the experimenter could ask them to write a short letter to another participant in response to a prompt that is either extremely personal (e.g., ask them to describe a difficult life experience or share a secret) or not personal at all (e.g., ask them to describe what they ate for breakfast), before exposing them to the manipulation [cf. ^85^]. Participants could then be exposed to video recordings of a confederate who expresses different levels of empathy.
2. In order to prime participants’ levels of empathy, the experimenter could ask them to read one or more letters they are told were written by another participant. Those letters could contain extremely personal information in which, say, the letter writers recount great hardships, or impassive information in which, say, the letter writers describe their recent meals [cf. ^86,87^]. Again, participants could then be exposed to video recordings of a confederate who expresses various levels of need.
3. Experiments could be conducted in which different levels of need and empathy are primed in different participants (need, empathy, or both), after which they are exposed to video recordings of confederates who express different levels of need and empathy.
4. After priming, participants could be exposed (using text, voice, or video) to a live confederate who has been trained to react to different participants with different levels of need and empathy.
5. When conversational bots are up to the task (which they might be by the time you read this paper), human confederates could be replaced by bots programmed to respond to different participants with different levels of need and empathy. Employing bots in this way might help to improve the consistency of responses.

We do not intend in this paper to imply that VTEB is in any sense an exhaustive theory. Emotional bonding, especially the extreme manifestation of it we call “love,” is indeed mysterious. In the movie “Interstellar,” one of the main characters, an astronaut and biologist, suddenly rhapsodizes about love, passionately exclaiming:

Love isn’t something that we invented. It’s observable, powerful. It has to mean something. We love people who have died. Where’s the social utility in that? Maybe it means something more, something we can’t yet understand. Maybe it’s some evidence, some artifact of a higher dimension that we can’t consciously perceive. I’m drawn across the universe to someone I haven’t seen in a decade, who I know is probably dead. Love is the one thing we’re capable of perceiving that transcends dimensions of time and space. Maybe we should trust that, even if we can’t understand it yet.

Emotional bonding is a complex process involving many factors—behavior, appearance, odor, voice characteristics, hormones, attachment styles, personality traits, gender, sexual orientation, and so on, not to mention people’s entire environmental histories. We would be naïve, if not impertinent, to suggest that VTEB has all the answers. That said, we believe that the study we have presented suggests that quantifying the role that need and empathy play in emotional bonding can enhance our understanding of the process and, we believe, does so in a way that might yield increasingly predictive models.

## Supplementary Information

Below is the link to the electronic supplementary material.

Supplementary Material 1

## Data Availability

An anonymized version of the data can be accessed at https://doi.org/10.5281/zenodo.17345002. Data can also be requested from the American Institute for Behavioral Research and Technology [info@aibrt.org]. The data have been anonymized to comply with requirements of the sponsoring institution’s Institutional Review Board (IRB).
