# Supplementary material for: Preliminary experimental support for a vulnerability theory of emotional bonding
Source: Sci Rep. 2025 Nov 17;15:40273. doi: 10.1038/s41598-025-24119-z (PMC12623801; doi:10.1038/s41598-025-24119-z)
Supplement: Supplementary file 1 — Supplementary Material 1 [file 41598_2025_24119_MOESM1_ESM.docx]

**Supplementary Information for:**

**Preliminary Experimental Support for a Vulnerability Theory of Emotional Bonding**

Robert Epstein^*^, Amanda Newland, & Camille Reid

**Supplementary Table S1**

*Processes that Strengthen Emotional Bonds According to Relationship Science (In Alphabetical Order), with Supporting Peer-Reviewed Studies*.

| ***1*. Accommodation*:** Research has found that accommodation is an important behavioral maintenance mechanism and it is a pro-relationship act that enhances trust between partners and increases willingness to become dependent on the relationship.  *References*: [1, 2, 3, 4] |
| --- |
| ***2. Agreement/Similarity*:** Attitude similarity is associated with compatibility, attraction, interconnectedness, marital quality, and relationship satisfaction. Similarity in personality traits as well as arbitrary similarities in names and birthdays are associated with increased attraction and higher marital quality.  *References*: [5, 6, 7, 8, 9, 10, 11, 12, 13, cf. 14] |
| ***3*. Alcohol*:** Alcohol has been shown to increase attractiveness ratings of strangers as well as reduce anxiety and make people feel bolder, allowing them to engage in sexual and courtship behaviors that they would otherwise be too nervous to try.  *References*: [15, 16, 17, 18, cf. 19] |
| ***4*. Appreciation/Gratitude*:** Research has shown that appreciation and gratitude promote relationship formation and maintenance. People who are appreciative of their partner have greater relationship satisfaction and are more committed to their relationships.  *References*: [20, 21, 22, 23, 24] |
| ***5*. Attention/Exclusivity*:** Research has shown that people in committed and loving relationships show less interest in and decreased attention to attractive alternatives.  *References*: [25, 26, 27, 28] |
| ***6*. Comfort/Emotional Support*:** Receiving emotional support from a partner is associated with increases in relationship satisfaction, feelings of caring, improved mood, relationship well-being, and oxytocin production.  *References*: [29, 30, 31, 32] |
| ***7*. Commitment*:** Researchers have found that high levels of commitment are associated with higher relationship stability, higher relationship satisfaction, and lower levels of vulnerability to negative partner characteristics.  *References*: [3, 33, 34, 35, 36, 37, 38] |
| ***8*. Communication*:** Positive and effective communication is associated with relationship well-being, relationship satisfaction, and relationship stability.  *References*: [39, 40, 41, 42, 43, cf. 44, 45] |
| ***9*. Compliments*:** Research has found that compliments can increase attraction and are a core strategy in relationship maintenance.  *References*: [46, 47, 48, 49] |
| ***10*. Coping with Illness*:** Dyadic coping has been found to be positively correlated with relationship satisfaction in couples where one partner has developed a chronic illness. Dyadic coping requires couples to view the illness of one person in the dyad as a shared interpersonal experience. Some strategies used in dyadic coping are a mutual sharing of personal experiences and developing a shared perception that the couple is “in it together.”  *References*: [35, 50, 51, 52, 53, 54, 55] |
| ***11*. Counseling*:** Many different marital counseling programs have been developed in the past few decades and some have shown to have moderate success at preventing or decreasing marital distress. 70% of couples who received Emotionally Focused Couples Therapy (EFT) were symptom free at the end of their treatment.  *References*: [42, 56, 57, 58, 59, 60, 61, 62] |
| ***12**. *Danger/Physiological Arousal*:** People in high states of arousal, such as fear, rejection, frustration, exercise, and sexual arousal, have been found to experience higher levels of attraction to novel faces or people and were more likely to express statements of love to their partner. Adventure, sense of risk, and other arousing emotions have also been shown to fuel romantic passion in couples.  *References*: [63, 64, 65, 66, 67, 68, 69, 70, 71] |
| ***13*. Deadlines/Pressure*:** Researchers have found that men and women in bars rate strangers more attractive the closer it is to closing time. It has also been found that people perceive themselves as more attractive later in the night, regardless of how much alcohol they had consumed.  *References*: [72, 73, 74] |
| ***14*. Emotional Intimacy*:** Emotional intimacy has been found to be associated with relationship quality, mental health, sexual desire, physical intimacy enjoyment, and attachment.  *References*: [75, 76, 77, 78] |
| ***15*. Encouragement/Support*:** Spousal and partner support has been found to be an important factor for attachment and promotes relationship satisfaction.  *References*: [79, 80, 81] |
| ***16. Familiarity*:** Familiarity with and repeated exposure to a person increases attractiveness and likeability ratings of that person.  *References*: [82, 83, 84, 85] |
| ***17*. Forbidden Activities*:** Some studies have found that couples that face parental interference or less network approval of their relationship can have increased feelings of commitment and romantic love.  *References*: [86, 87, cf. 88] |
| ***18*. Forgiveness*:** Research has found that forgiveness is an important behavioral maintenance mechanism and it is a mediator between adult attachment and marital satisfaction. Research has also found that for people who are extremely close with and are more committed to their partners, forgiveness is a more automatic process and strongly linked to well-being.  *References*: [3, 89, 90, 91] |
| ***19*. Gazing*:** Gazing and eye-contact has been found to increase feelings of liking, attraction, and love.  *References*: [92, 93, 94, 95] |
| ***20*. Humor*:** Both men and women are attracted to a good sense of humor. Humor is a significant predictor of mating success and number of sexual partners in a lifetime. In long-term relationships, humor has been found to be an important mechanism in building relationships.  *References*: [96, 97, 98, 99] |
| ***21*. Kindness*:** Globally, both men and women report kindness to be a necessary quality for mate selection.  *References*: [100, 101, 102] |
| ***22. Mimicking*:** Researchers have found that being mimicked increases liking for the mimicker.  *References*: [103, 104, 105, 106] |
| ***23*. Novelty/Change/Play*:** Research has found that adventurous, risky, novel, and exciting activities increase intimacy, romantic passion, sexual desire, feelings of security, and relationship satisfaction.  *References*: [70, 107, 108, 109, 110, 111] |
| ***24*. Physical Intimacy*:** Frequent and satisfying physical intimacy has been shown to promote passionate love, predict sexual and romantic satisfaction, and increase oxytocin levels, which has been long linked to emotional bonding.  *References*: [32, 63, 112, 113, 114, 115] |
| ***25. Positive Illusions/Idealization*:** Exaggerated positive views and idealization of a partner is associated with marital satisfaction and commitment.  *References*: [116, 117, 118, 119, cf. 120] |
| ***26*. Protection*:** Evolutionary psychologists have repeatedly found that the ability to offer protection is an attractive quality in a potential mate for women. Men’s musculature and willingness to invest in and protect offspring have both been found to be crucial components in male attractiveness. It has also been found that loss of protection is considered a major loss in the event of a breakup.  *References:* [121, 122, 123, 124] |
| ***27. Proximity*:** The less physical distance there is between two people the higher the chance is that they will develop a social connection. Less physical distance between couples is also associated with reduced attachment anxiety.  *References*: [125, 126, 127, 128, 129] |
| ***28. Resources/Wealth/Status*:** Income and wealth are important qualities for mate preference for women across the world.  *References*: [100**,** 102, 130**,** 131, 132, 133] |
| ***29*. Sacrifice*:** Research has found that when partners are motivated to sacrifice for their partner’s well-being or happiness, they themselves experience higher levels of personal well-being and satisfaction with their relationship. Researchers have also found that when people perceive their partner’s sacrifice, they feel greater appreciation for their partner and greater relationship satisfaction.  *References*: [35, 134, 135, 136, cf. 137] |
| ***30*. Self-Disclosure*:** Self-disclosure has been found to increase marital satisfaction, feelings of attachment and closeness, and passionate love. Researchers have also found that people who engage in self-disclosure are more well liked than those who don’t. Self-disclosure that is vulnerable or intimate has a higher positive impact on relationships than general self-disclosures.  *References*: [138, 139, 140, 141, 142, 143, 144, 145, 146, 147] |
| ***31. Sharing Activities*:** Sharing activities has found to be an effective relationship maintenance strategy for both friendships and romantic relationships. Engaging in new and fun activities with a partner increases social closeness and relationship quality.  *References*: [148, 149, 150, 151, 152] |
| ***32. Social Support*:** Social support for a relationship from parents, family, and friends is associated with greater relationship well-being and relationship stability.  *References*: [88, 153, 154, 155, 156, 157] |
| ***33*. Sympathy/Empathy/Understanding*:** Higher empathy and understanding from a partner is related to relationship satisfaction and positive relationship outcomes.  *References*: [158, 159, 160, 161, 162] |
| ***34. Thoughtfulness*:** Research has found that the receipt of thoughtful benefits increases relationship connection and satisfaction.  *References*: [163, 164] |
| ***35*. Tragedy/Loss*:** Studies have found that couples who share their grief or experience a tragedy, such as a natural disaster, are sometimes brought closer by their shared experience. Researchers have also found that veterans who were in heavy combat bonded more than those who were not. They also found that the loss of a significant other resulted in more intense and long-lasting postwar relationships.  *References:* [165, 166, 167, 168] |
| ***36. Vacations*:** Research has found that vacations help maintain and improve romantic relationships.  *References*: [169, 170] |

*Examples of areas of emotional bonding research that can reasonably be interpreted from a vulnerability perspective.

**Supplementary Text S1**

*Other Relevant Research on Emotional Bonding*.

It is beyond the scope of this paper to review the extensive literature on factors that increase emotional bonding [although see 171, 172, 173]. We have summarized 36 relatively distinct areas of applicable research in Table S1 (above). Most, but not all, of these areas of research are, in our view, studying vulnerability, at least to some extent. For example, research on sacrifice in relationships shows that when one partner makes a sacrifice for the other, that sacrifice tends to lead to greater partner appreciation and higher ratings of relationship quality from both partners, especially when that sacrifice was made with approach motives [134, 136]. When one partner is offered a job in a different state and the other partner gives up (sacrifices) his or her own job and social network to accompany his or her partner, the vulnerability – in this case, mutual – is apparent. Partner A needs support from Partner B, who empathizes with that need and agrees to accompany him or her. Partner B now needs support from Partner A, who, recognizing the sacrifice A is making, supports and shows gratitude for A’s sacrifice (thus showing empathy). Both parties are weakened in this situation (one meaning of the word “vulnerable”), and both are strengthened by the support of the other.

Also relevant are studies on physical and emotional intimacy. A study of 120 new lovers found that the oxytocin levels in the couples were correlated with their frequency of affectionate touch [32]. Oxytocin is often referred to as “the love hormone” for the role it plays in social bonding [174]. Mutual vulnerability is obvious whenever voluntary physical intimacy occurs. Each person expresses a physical need, and each satisfies the other’s need (one way of demonstrating empathy). In the case of rape, the vulnerability is distorted. The rapist seeks to satisfy his or her need but does so by forcing the victim to satisfy that need. As a result of the attack, the victim now has new needs (for protection, escape, and comfort), but the rapist satisfies none of these needs. In other words, voluntary physical intimacy will generally strengthen emotional bonds [78, 175, 176]; involuntary physical intimacy generally damages or destroys such bonds [177].

Sometimes the role that vulnerability plays in human interactions is hidden, or at least not evident at first glance. Such interactions can greatly strengthen emotional bonds, however. Mutual eye gazing, for example, can rapidly cause total strangers to bond emotionally [92, 94, 95], even though gazing appears at first glance to be a benign activity. The vulnerability becomes evident when one draws the distinction between gazing and *staring*. For many mammals, staring is a threat gesture [178, 179, 180, 181]. This is as true for humans as it is for lizards and monkeys; first-time visitors to New York City are often warned, for example, to avoid locking eyes with strangers when riding the subways [182].

So how is gazing different? When two people – even strangers, as we noted – allow each other to gaze continuously into each other’s eyes, they are making themselves as vulnerable to each other as if they have just disrobed. They are allowing themselves to demonstrate their weakness, choosing to interpret the other person’s gaze not as a threat but as a sign of interest. People who allow such an interaction to occur often report increased feelings of attraction, intimacy, and trust [94, 183, 184]. The eye-gazing exercise is also sometimes referred to by psychologists and couples therapists as “soul gazing,” consistent with the adage that “the eyes are the windows of the soul” [185, cf. 126].

Perhaps the most dramatic example of an interaction that appears at first to be irrelevant to vulnerability is commitment. In a study of how love emerged in the arranged marriages of 22 people from six countries [35], of the 36 different types of interactions that were examined in the study, commitment emerged as one of the two strongest predictors of emotional bonding (the other was sacrifice). (Note that this was a survey study, not an experimental study, so no causal relationship is implied.) But what role does vulnerability play in commitment?

In our view, mutual commitment – at least strong mutual commitment of the sort that might be expressed during a wedding ceremony – conceivably entails a higher degree of vulnerability than any other human interaction. Each party is expressing boundless levels of both weakness and empathy, promising to support the other person no matter how bad things get: “for better or for worse, for richer or for poorer, in sickness and in health, ‘til death do us part.” In cultures where that kind of commitment is expressed, the moment such pledges are exchanged might be the most vulnerable moment each partner will ever experience.

We would be remiss if we did not mention that vulnerability has become an iconic topic on the internet and in pop culture. Interest was driven in part by a highly popular TED talk by University of Houston social work professor Brené Brown, which, at this writing (May 22, 2025), has been viewed online more than 67 million times on TED’s website [186]. “Vulnerability,” said Brown in the video, “is the core of shame and fear and our struggle for worthiness, but it appears that it’s also the birthplace of joy, of creativity, of belonging, of love.” To add meaning to our lives, she said, we must “let ourselves be seen, deeply seen, vulnerably seen… to love with our whole hearts.” She elaborated on this idea in *Daring Greatly: How the Courage to be Vulnerable Transforms the Way We Live, Love, Parent and Lead*, a best-selling book [187]. Although this book refers to Brown’s “research,” by that she seems to mean her own interpretations of interviews she conducted with more than a thousand people. None of this research was subjected to peer review, as far as we can tell.

The strong public interest in vulnerability has led to additional thinking about vulnerability from a behavioral perspective [e.g., 188], the role that vulnerability training can play in marital and couples therapy [e.g., 189, cf. 190, 191], and other applications [192, 193, 194, 195].

**Supplementary Text S2**

*Supplementary References*.

1. Overall, N. C. & Sibley, C. G. When accommodation matters: Situational dependency within daily interactions with romantic partners. *J. Exp. Soc. Psychol*. **44**, 95-104; <https://doi.org/10.1016/j.jesp.2007.02.005> (2008).
2. Rusbult, C. E., Bissonnette, V. L., Arriaga, X. B. & Cox, C. L. Accommodation processes during the early years of marriage in *The developmental course of marital dysfunction* (ed. Bradbury, T. N.) 74-113 <https://doi.org/10.1017/CBO9780511527814> (Cambridge University Press, 1998).
3. Rusbult, C. E., Olsen, N., Davis, J. L. & Hannon, M. A. Commitment and relationship maintenance mechanisms in *Close romantic relationships: Maintenance and enhancement* (ed. Harvey, J. H. & Wenzel, A. E.) 87-113 (Lawrence Erlbaum Associates, 2001).
4. Wieselquist, J., Rusbult, C. E., Foster, C. A. & Agnew, C. R. Commitment, pro-relationship behavior, and trust in close relationships. *J. Pers. Soc. Psychol*. 77, 942-966; <https://doi.org/10.1037/0022-3514.77.5.942> (1999).
5. Byrne, D. & Clore, G. L. A reinforcement model of evaluation responses in *Foundations of interpersonal attraction* (ed. Huston, T. L.) 143-170 (Academic Press, 1970).
6. Byrne, D. & Nelson, D. The effect of topic importance and attitude similarity-dissimilarity on attraction in a multistranger design. *Psychon. Sci.* **3**, 449-450; <https://doi.org/10.3758/BF03343226> (1965).
7. Cheon, J. E. & Kim, Y.-H. Similarity as a safe haven: Similarity leads to satisfaction in prevention focus. *J. Soc. Pers. Relatsh*. **41**, 69-90; <https://doi.org/10.1177/02654075231210851> (2024).
8. He, J., Zhao, Y., Chen, B., Bao, Y. & Xiao, Z. Similarity enhances psychological compatibility: Serial mediation effect of psychological kinship and intergroup contact. *Heliyon*, **10**, e36262; <https://doi.org/10.1016/j.heliyon.2024.e36262> (2024).
9. Jones, J. T., Pelham, B. W., Carvallo, M. & Mirenberg, M. C. How do I love thee? Let me count the js: Implicit egotism and interpersonal attraction. *J. Pers. Soc. Psychol.* **87**, 665-683; <https://doi.org/10.1037/0022-3514.87.5.665> (2004).
10. McPherson, M., Smith-Lovin, L. & Cook, J. M. Birds of a feather: Homophily in social networks. *Annu. Rev. Sociol.* **27**, 415-444; <https://www.jstor.org/stable/2678628> (2001).
11. Peacock, C. & Pederson, J. R. Love and politics: The influence of politically (dis)similar romantic relationships on political participation and relationship satisfaction. *Hum. Commun. Res.* **48**, 567-578; <https://doi.org/10.1093/hcr/hqac011> (2022).
12. Singh, R. *et al.* Attitude similarity and attraction: Validation, positive affect, and trust as sequential mediators. *Pers. Relatsh*. **24**, 203-222; <https://doi.org/10.1111/pere.12178> (2017).
13. Watson, D. *et al.* Match makers and deal breakers: Analyses of assortative mating in newlywed couples. *J. Pers*. **72**, 1029-1068; <https://doi.org/10.1111/j.0022-3506.2004.00289.x> (2004).
14. Humberg, S., Gerlach, T. M., Franke-Prasse, T., Geukes, K. & Back, M. D. Is (actual or perceptual) personality similarity associated with attraction in initial romantic encounters? A dyadic response surface analysis. *Pers. Sci.* **4**, <https://doi.org/10.5964/ps.7551> (2023).
15. George, W. H. & Stoner, S. A. Understanding acute alcohol effects on sexual behavior. *Annu. Rev. Sex Res.* **11**, 92-124; <http://dx.doi.org/10.1080/10532528.2000.10559785> (2000).
16. Lyvers, M., Cholakians, E., Puorro, M. & Sundram, S. Beer goggles: Blood alcohol concentration in relation to attractiveness ratings for unfamiliar opposite sex faces in naturalistic settings. *J. Soc. Psychol*. **151**, 105-112; <https://doi.org/10.1080/00224540903366776> (2011).
17. Mutchler, M. G., McDavitt, B. & Gordon, K. K. "Becoming Bold": Alcohol use and sexual exploration among Black and Latino young men who have sex with men (YMSM). *J. Sex Res.* **51**, 696-710; <https://doi.org/10.1080/00224499.2013.772086> (2013).
18. Steele, C. M. & Josephs, R. A. Alcohol myopia: Its prized and dangerous effects. *Am. Psychol.* **45**, 921-933; <https://doi.org/10.1037/0003-066X.45.8.921> (1990).
19. Toates, F. An integrative theoretical framework for understanding sexual motivation, arousal, and behavior. *J. Sex Res.* **46**, 168-193; <https://doi.org/10.1080/00224490902747768> (2009).
20. Algoe, S. B., Haidt, J. & Gable, S. L. Beyond reciprocity: gratitude and relationships in everyday life. *Emotion*. **8**, 425-429; <https://doi.org/10.1037/1528-3542.8.3.425> (2008).
21. Barton, A. W. & Gong, Q. A ‘thank you’ really would be nice: Perceived gratitude in family relationships. *J. Posit. Psychol.* 1-10; <https://doi.org/10.1080/17439760.2024.2365472> (2024).
22. Berger, A. R. & Janoff-Bulman, R. Costs and satisfaction in close relationships: The role of loss–gain framing. *Pers. Relatsh.* **13**, 53-68; <https://doi.org/10.1111/j.1475-6811.2006.00104.x> (2006).
23. Floyd, K. Communicating affection: Interpersonal behavior and social context. *Cambridge University Press*. (2006).
24. Gordon, A. M., Impett, E. A., Kogan, A., Oveis, C. & Keltner, D. To have and to hold: gratitude promotes relationship maintenance in intimate bonds. *J. Pers. Soc. Psychol.* **103**, 257-274; <https://doi.org/10.1037/a0028723> (2012).
25. Freeman, H. *et al.* I only have eyes for you: Oxytocin administration supports romantic attachment formation through diminished interest in close others and strangers. *Psychoneuroendocrinology*. **134**, 105415; <https://doi.org/10.1016/j.psyneuen.2021.105415> (2021).
26. Gonzaga, G. C., Haselton, M. G., Smurda, J., Davies, M. S. & Poore, J. C. Love, desire, and the suppression of thoughts of romantic alternatives. *Evol. Hum. Behav.* **29**, 119-126; <https://doi.org/10.1016/j.evolhumbehav.2007.11.003> (2008).
27. Miller, R. S. Attending to temptation: The operation (and perils) of attention to alternatives in close relationships in *Social relationships: Cognitive, affective, and motivational processes* (Eds. Forgas, J. P. & Fitness, J.) 321-337 (Psychology Press, 2008).
28. Visserman, M. L. & Karremans, J. C. Romantic relationship status biases the processing of an attractive alternative's behavior. *Pers. Relatsh.* **21**, 324-334; <https://doi.org/10.1111/pere.12035> (2014).
29. Austin, K. W., Kane, H. S. & Ackerman, R. A. Emotional approach coping and daily support behavior in romantic relationships. *J. Soc. Pers. Relatsh.* **39**, 526-548; <https://doi.org/10.1177/02654075211041657> (2021).
30. Collins, N. L. & Feeney, B. C. A safe haven: An attachment theory perspective on support seeking and caregiving in intimate relationships. *J. Pers. Soc. Psychol.* **78**, 1053-1073; <https://doi.org/10.1037/0022-3514.78.6.1053> (2000).
31. Lorenzo, J. M., Barry, R. A. & Khalifian, C. E. More or less: Newlyweds’ preferred and received social support, affect, and relationship satisfaction. *J. Fam. Psychol.* **32**, 860-872; <https://doi.org/10.1037/fam0000440> (2018).
32. Schneiderman, I., Zagoory-Sharon, O., Leckman, J. F. & Feldman, R. Oxytocin during the initial stages of romantic attachment: Relations to couples’ interactive reciprocity. *Psychoneuroendocrinology*. **37**, 1277-1285; <https://doi.org/10.1016/j.psyneuen.2011.12.021> (2012).
33. Arriaga, X. B., Slaughterbeck, E. S., Capezza, N. M. & Hmurovic, J. L. From bad to worse: Relationship commitment and vulnerability to partner imperfections. *Pers. Relatsh.* **14**, 389-409; <https://doi.org/10.1111/j.1475-6811.2007.00162.x> (2007).
34. Cate, R. M., Levin, L. A. & Richmond, L. S. Premarital relationship stability: A review of recent research. *J. Soc. Pers. Relatsh.* **19**, 261-284; <https://doi.org/10.1177/0265407502192005> (2002).
35. Epstein, R., Pandit, M. & Thakar, M. How love emerges in arranged marriages: Two cross-cultural studies. *J. Comp. Fam. Stud*. **44**, 341-360; <https://doi.org/10.3138/jcfs.44.3.341> (2013).
36. Etcheverry, P. E. & Le, B. Thinking about commitment: Accessibility of commitment and prediction of relationship persistence, accommodation, and willingness to sacrifice. *Pers. Relatsh.* **12**, 103-123; <https://doi.org/10.1111/j.1350-4126.2005.00104.x> (2005).
37. Ito, K., Yang, S. & Li, L. M. W. Changing Facebook profile pictures to dyadic photos: Positive association with romantic partners' relationship satisfaction via perceived partner commitment. *Comput. Hum. Behav.* **120**, 106748; <https://doi.org/10.1016/j.chb.2021.106748> (2021).
38. Lydon, J. E., Fitzsimons, G. M. & Naidoo, L. Devaluation versus enhancement of attractive alternatives: A critical test using the calibration paradigm. *Pers. Soc. Psychol. Bull.* **29**, 349-359; <https://doi.org/10.1177/0146167202250202> (2003).
39. Carton, J. S., Kessler, E. A. & Pape, C. L. Nonverbal decoding skills and relationship well-being in adults. *J. Nonverbal Behav.* **23**, 91-100; <https://doi.org/10.1023/A:1021339410262> (1999).
40. Gottman, B. A. & Delaney, H. J. Gender differences in perception of attractiveness of men and women in bars. *Pers. Soc. Psychol. Bull.* **16**, 378-391; <https://doi.org/10.1177/0146167290162017>
41. MacNeil, S. & Byers, E. S. Dyadic assessment of sexual self-disclosure and sexual satisfaction in heterosexual dating couples. *J. Soc. Pers. Relatsh.* **22**, 169-181; <https://doi.org/10.1177/0265407505050942> (2005).
42. Markman, H., Stanley, S. & Blumberg, S. L. *Fighting for your marriage: Positive steps for preventing divorce and preserving a lasting love*. (Jossey-Bass, 1994).
43. Sprecher, S. & Cate, R. M. Sexual satisfaction and sexual expression as predictors of relationship satisfaction and stability in *The handbook of sexuality in close relationships* (eds. Harvey, J. H., Wenzel, A. & Sprecher, S.) 235-256 (Lawrence Erlbaum Associates, 2004).
44. Johnson, M. D. *et al.* Within-couple associations between communication and relationship satisfaction over time. *Pers. Soc. Psychol. Bull.* **48**, 534-549; <https://doi.org/10.1177/01461672211016920> (2022).
45. Lavner, J. A., Karney, B. R. & Bradbury, T. N. Does couples' communication predict marital satisfaction, or does marital satisfaction predict communication? *J. Marriage Fam*. **78**, 680-694; <https://doi.org/10.1111/jomf.12301> (2016).
46. Brumbaugh, C. C., Baren, A. & Agishtein, P. Attraction to attachment insecurity: Flattery, appearance, and status's role in mate preferences. *Pers. Relatsh*. **21**, 288-308; <https://doi.org/10.1111/pere.12032> (2014).
47. Canary, D. J. & Stafford, L. Relational maintenance strategies and equity in marriage. *Commun. Monogr*. **59**, 243-267; <https://doi.org/10.1080/03637759209376268> (1992).
48. Honeycutt, J. M. & Patterson, J. Affinity strategies in relation-ships: The role of gender and imagined interactions in maintaining liking among college roommates. *Pers. Relatsh.* **4**, 35-46; <https://doi.org/10.1111/j.1475-6811.1997.tb00129.x> (2005).
49. Hu, Y., Ogolsky, B. G. & Stafford, L. The role of individual- and interactive-level relationship maintenance on married couples' commitment. *Pers. Relatsh*. **30**, 1426-1448; <https://doi.org/10.1111/pere.12517> (2023).
50. Bertschi, I. C., Meier, F. & Bondenmann, G. Disability as an interpersonal experience: A systematic review on dyadic challenges and dyadic coping when one partner has a chronic physical or sensory impairment. *Front. Psychol*. **12**, <https://doi.org/10.3389/fpsyg.2021.624609> (2021).
51. Falconier, M. K., Jackson, J. B., Hilpert, P. & Bodenmann, G. Dyadic coping and relationship satisfaction: a meta-analysis. *Clin. Psychol. Rev*. **42**, 28-46; <https://doi.org/10.1016/j.cpr.2015.07.002> (2015).
52. Ghafari, S., Khoshknab, M. F., Norouzi, K. & Mohamadi, E. Spousal support as experienced by people with multiple sclerosis: a qualitative study. *J. Neurosci. Nurs. J. Am. Assoc. Neurosci. Nurses*. **46**, E15-E24; <https://doi.org/10.1097/JNN.0000000000000081> (2014).
53. Lehane, C. M., Elsass, P., Hovaldt, H. B. & Dammeyer, J. A relationship-focused investigation of spousal psychological adjustment to dual-sensory loss. *Aging Ment. Health*. **22**, 397-404; <https://doi.org/10.1080/13607863.2016.1268091> (2016).
54. Traa, M. J., Vries, J. D., Bodenmann, G. & Oudsten, B. L. D. Dyadic coping and relationship functioning in couples coping with cancer: a systematic review. *Br. J. Health Psychol.* **20**, 85-114; <https://doi.org/10.1111/bjhp.12094> (2014).
55. Zhaoyang, R., Martire, L. M. & Stanford, A. M. Disclosure and holding back: Communication, psychological adjustment, and marital satisfaction among couples coping with osteoarthritis. *J. Fam. Psychol.* **32**, 412-418; <https://doi.org/10.1037/fam0000390> (2018).
56. Baucom, D. H., Epstein, N. & Stanton, S. The treatment of relationship distress: Theoretical perspectives and empirical findings in *The Cambridge handbook of personal relationships* (eds. Vangelisti, A. & Perlman, D.) 745-769 (Cambridge University Press, 2006).
57. Baucom, D. H., Hahlweg, K., Atkins, D. C., Engl, J. & Thurmaier, F. Long-term prediction of marital quality following a relationship education program: Being positive in a constructive way. *J. Fam. Psychol*. **20**, 448-455; <https://doi.org/10.1037/0893-3200.20.3.448> (2006).
58. Carroll, J. S. & Doherty, W. J. Evaluating the effectiveness of premarital prevention programs: A meta-analytic review of outcome research. *Fam. Relat*. **52**, 105-118; <https://doi.org/10.1111/j.1741-3729.2003.00105.x> (2004).
59. Freedman, C. M., Low, S. M., Markman, H. J. & Stanley, S. M. Equipping couples with the tools to cope with predictable and unpredictable crisis events: the PREP Program. *INTJEM*. **4**, 49-55 (2002).
60. Johnson, S. M. *The practice of emotionally focused couple therapy* (2nd ed.) <https://doi.org/10.4324/9780203843871> (Routledge, 2004).
61. Monarch, N. D., Hartman, S. G., Whitton, S. W. & Markman, H. J. The role of clinicians in the prevention of marital distress and divorce in *A clinician’s guide to maintaining and enhancing close relationships* (eds. Harvey, J. H. & Wenzel, A.) 233-258 (Routledge, 2002).
62. Spengler, P. M., Lee, N. A., Wiebe, S. A. & Wittenborn, A. K. A comprehensive meta-analysis on the efficacy of emotionally focused couple therapy. *Couple Fam. Psychol.: Res. Pract.* **13**, 81-99; <https://doi.org/10.1037/cfp0000233> (2024).
63. Berscheid, E. & Hatfield, E. A little bit about love: A minor essay on a major topic in *Foundations of interpersonal attraction* (ed. Huston, T.) 355-381 <https://doi.org/10.1016/C2013-0-10883-5> (Academic Press, 1974).
64. Dutton, D. G. & Aron, A. P. Some evidence for heightened sexual attraction under conditions of high anxiety. *J. Pers. Soc. Psychol.* **30**, 510-517; <https://doi.org/10.1037/h0037031> (1974).
65. Bastian, B., Jetten, J. & Ferris, L. J. Pain as social glue: Shared pain increases cooperation. *Psychol. Sci.* **25**, 2079-2085; <https://doi.org/10.1177/0956797614545886> (2014).
66. Demiroglu, S. *et al*. Shared suffering predicts prosocial commitment among Turkish earthquake survivors. *Sci. Rep*. **15**, 8543; <https://doi.org/10.1038/s41598-025-90921-4> (2025).
67. Dermer, M. & Pyszczynski, T. A. Effects of erotica upon men's loving and liking responses for women they love. J*. Pers. Soc. Psychol.* **36**, 1302-1309; <https://doi.org/10.1037/0022-3514.36.11.1302> (1978).
68. Foster, C. A., Witcher, B. S., Campbell, W. K. & Green, J. D. Arousal and attraction: Evidence for automatic and controlled processes. *J. Pers. Soc. Psychol*. **74**, 86-101; <https://doi.org/10.1037/0022-3514.74.1.86> (1998).
69. Han, S., Gao, J., Xing, W., Zhou, X. & Luo, Y. Facial attractiveness in the eyes of men with high arousal. *Brain Behav*. **13**, e3132; <https://doi.org/10.1002/brb3.3132> (2023).
70. Mitchell, S. A. *Can love last? The fate of romance over time*. (W. W. Norton & Company, 2002).
71. White, G. L., Fishbein, S. & Rutsein, J. Passionate love and the misattribution of arousal. *J. Pers. Soc. Psychol*. **41**, 56-62; <https://doi.org/10.1037/0022-3514.41.1.56> (1981).
72. Pennebaker, J. W. *et al*. Don’t the girls’ get prettier at closing time: A country and western application to psychology. *Pers. Soc. Psychol. Bull.* **5**, 122-125; <https://doi.org/10.1177/014616727900500127> (1979).
73. Gladue, B. A. & Delaney, H. J. Gender differences in perception of attractiveness of men and women in bars. *Pers. Soc. Psychol. Bull.* **16**, 378-391; <https://doi.org/10.1177/0146167290162017> (1990).
74. Otterbring, T. & Rolschau, K. Beauty is in the eye of the beer holder but rarely because of the beer. *Pers. Individ. Differ*. **179**, <https://doi.org/10.1016/j.paid.2021.110921> (2021).
75. Bauminger, N., Finzi-Dottan, R., Chason, S. & Har-Even, D. Intimacy in adolescent friendship: The roles of attachment, coherence, and self-disclosure. *J. Soc. Pers. Relatsh*. **25**, 409-428; <https://doi.org/10.1177/0265407508090866> (2008).
76. Frost, D. M. The narrative construction of intimacy and affect in relationship stories: Implications for relationship quality, stability, and mental health. *J. Soc. Pers. Relatsh*. **30**, 247-269; <https://doi.org/10.1177/0265407512454463> (2012).
77. Shrier, L. A. & Blood, E. A. Momentary desire for sexual intercourse and momentary emotional intimacy associated with perceived relationship quality and physical intimacy in heterosexual emerging adult couples. *J. Sex Res*. **53**, 968-978; <https://doi.org/10.1080/00224499.2015.1092104> (2016).
78. van Lankveld, J. J. D. M., Dewitte, M., Verboon, P., van Hooren, S. A. H. Associations of intimacy, partner responsiveness, and attachment-related emotional needs with sexual desire. *Front. Psychol*. **12**, <https://doi.org/10.3389/fpsyg.2021.665967> (2021).
79. Feeney, B. C. & Collins, N. L. The importance of relational support for attachment and exploration needs. *Curr. Opin. Psychol*. **25**, 182-186; <https://doi.org/10.1016/j.copsyc.2018.11.011> (2019).
80. Jakubiak, B. K. & Feeney, B. C. Daily goal progress is facilitated by spousal support and promotes psychological, physical, and relational well-being throughout adulthood. *J. Pers. Soc. Psychol*. **111**, 317-340; <https://doi.org/10.1037/pspi0000062> (2016).
81. Lemay, E. P., Jr., Clark, M. S. & Feeney, B. C. Projection of responsiveness to needs and the construction of satisfying communal relationships. *J. Pers. Soc. Psychol*. **92**, 834-853; <https://doi.org/10.1037/0022-3514.92.5.834> (2007).
82. Moreland, R. L. & Beach, S. R. Exposure effects in the classroom: The development of affinity among students. *J. Exp. Soc. Psychol*. **28**, 255-276; <https://doi.org/10.1016/0022-1031(92)90055-O> (1992).
83. Moreland, R. L. & Zajonc, R. B. Exposure effects in person perception: Familiarity, similarity, and attraction. *J. Exp. Soc. Psychol*. **18**, 395-415; <https://doi.org/10.1016/0022-1031(82)90062-2> (1982).
84. Reis, H. T., Maniaci, M. R., Caprariello, P. A., Eastwick, P. W. & Finkel, E. J. Familiarity does indeed promote attraction in live interaction. *J. Pers. Soc. Psychol*. **101**, 557-570; <https://doi.org/10.1037/a0022885> (2011).
85. Zajonc, R. B. Mere exposure: A gateway to the subliminal. *Curr. Dir. Psychol. Sci*. **10**, 224-228; <https://doi.org/10.1111/1467-8721.00154> (2001).
86. Driscoll, R., Davis, K. E. & Lipetz, M. E. Parental interference and romantic love: The Romeo and Juliet effect. *J. Pers. Soc. Psychol.* **24**, 1-10; <https://doi.org/10.1037/h0033373> (1972).
87. Besikci, E., Agnew, C. R. & Yildirim, A. It's my partner, deal with it: Rejection sensitivity, normative beliefs, and commitment. *Pers. Relatsh*. **23**, 384-395; <https://doi.org/10.1111/pere.12131> (2016).
88. Sinclair, H. C., Hood, K. B. & Wright, B. L. Revisiting the Romeo and Juliet Effect (Driscoll, Davis, & Lipetz, 1972): Reexamining the links between social network opinions and romantic relationship outcomes. *Soc. Psychol*. **45**, 170-178; <https://doi.org/10.1027/1864-9335/a000181> (2014).
89. Bono, G., McCullough, M. & Root, L. M. Forgiveness, feeling connected to others, and well-being: Two longitudinal studies. *Pers. Soc. Psychol. Bull*. **34**, 182-195; <https://doi.org/10.1177/0146167207310025> (2007).
90. Chung, M.-S. Pathways between attachment and marital satisfaction: The mediating roles of rumination, empathy, and forgiveness. *Pers. Individ. Differ*. **70**, 246-251; <https://doi.org/10.1016/j.paid.2014.06.032> (2014).
91. Karremans, J. C. & Aarts, H. The role of automaticity in determining the inclination to forgive close others. *J. Exp. Soc. Psychol*. **43**, 902-917; <https://doi.org/10.1016/j.jesp.2006.10.012> (2007).
92. Hoffmann, A., Schiestl, S., Sinske, P., Gondan, M., Sachse, P. & Maran, T. Sharing and receiving eye-contact predicts mate choice after a 5-minute conversation: evidence from a speed-dating study. *Arch. Sex. Behav*. **53**, 959-968; <https://doi.org/10.1007/s10508-023-02806-0> (2024).
93. Kampe, K. K. W., Frith, C. D., Dolan, R. J. & Frith, U. Reward value of attractiveness and gaze. *Nature*. **413**, 589; <https://doi.org/10.1038/35098149> (2001).
94. Kellerman, J., Lewis, J. & Laird, J. D. Looking and loving: The effects of mutual gaze on feelings of romantic love. *J. Res. Pers*. **23**, 145-161; <https://doi.org/10.1016/0092-6566(89)90020-2> (1989).
95. Mason, M. F., Tatkow, E. P. & Macrae, C. N. The look of love: Gaze shifts and person perception. *Psychol. Sci*. **16**, 236-239; <https://doi.org/10.1111/j.0956-7976.2005.00809.x> (2005).
96. Greengross, G. & Miller, G. Humor ability reveals intelligence, predicts mating success, and is higher in males. *Intelligence*. **39**, 188-192; <https://doi.org/10.1016/j.intell.2011.03.006> (2011).
97. Jach, L., Kubicius, D. & Jonason, P. K. “Do they fit together like the Joker and Harley Quinn?”: Joking, laughing, humor styles, and dyadic adjustment among people in long-term romantic relationships. *Pers. Individ. Diff*. **199**, <https://doi.org/10.1016/j.paid.2022.111859> (2022).
98. Lauer, R. H., Lauer, J. C. & Kerr, S. T. The long-term marriage: Perceptions of stability and satisfaction. *Int. J. Aging Hum. Dev*. **31**, 189-195; <https://doi.org/10.2190/H4X7-9DVX-W2N1-D3BF> (1990).
99. Li, N. P. *et al*. An Evolutionary Perspective on Humor: Sexual Selection or Interest Indication? *Pers. Soc. Psychol. Bull*. **35**, 923-936; <https://doi.org/10.1177/0146167209334786> (2009).
100. Li, N. P., Bailey, J. M., Kenrick, D. T. & Linsenmeier, J. A. W. The necessities and luxuries of mate preferences: Testing the tradeoffs. *J. Pers. Soc. Psychol*. **82**, 947-955; <https://doi.org/10.1037/0022-3514.82.6.947> (2002).
101. Takayanagi, J. F. G. B., Siqueira, J. D., Silveira, P. S. P. & Valentova, J. V. What do different people look for in a partner? Effects of sex, sexual orientation, and mating strategies on partner preferences. *Arch. Sex. Behav*. **53**, 981-1000; <https://doi.org/10.1007/s10508-023-02767-4> (2024).
102. Walter, K. V. *et al*. Sex differences in mate preferences across 45 countries: A large-scale replication. *Psychol. Sci.* **31**, 408-423; <https://doi.org/10.1177/0956797620904154> (2020).
103. Bailenson, J. N. & Yee, N. Digital chameleons: Automatic assimilation of nonverbal gestures in immersive virtual environments. *Psychol. Sci*. **16**, 814-819; <https://doi.org/10.1111/j.1467-9280.2005.01619.x> (2005).
104. Chartrand, T. L. & Bargh, J. A. The chameleon effect: The perception–behavior link and social interaction. *J. Pers. Soc. Psychol*. **76**, 893-910; <https://doi.org/10.1037/0022-3514.76.6.893> (1999).
105. Duffy, K. A. & Chartrand, T. L. Mimicry: causes and consequences. *Curr. Opin. Behav. Sci*. **3**, 112-116; <https://doi.org/10.1016/j.cobeha.2015.03.002> (2015).
106. Kulesza, W. *et al*. Imagining is not observing: The role of simulation processes within the mimicry-liking expressway. *J. Nonverbal Behav*. **46**, 233-246; <https://doi.org/10.1007/s10919-022-00399-1> (2022).
107. Aron, A., Norman, C. C., Aron, E. N., McKenna, C. & Heyman, R. E. Couples' shared participation in novel and arousing activities and experienced relationship quality. *J. Pers. Soc. Psychol*. **78**, 273-284; <https://doi.org/10.1037//0022-3514.78.2.273> (2000).
108. Baumeister, R. F. & Bratslavsky, E. Passion, intimacy, and time: Passionate love as a function of change in intimacy. *Pers. Soc. Psychol. Rev*. **3**, 49-67; <https://doi.org/10.1207/s15327957pspr0301_3> (1999).
109. Cortes, K., Britton, E., Holmes, J. G. & Scholer, A. A. Our adventures make me feel secure: Novel activities boost relationship satisfaction through felt security. *J. Exp. Soc. Psychol*. **89**, 103992; <https://doi.org/10.1016/j.jesp.2020.103992> (2020).
110. Coulter, K. & Malouff, J. M. Effects of an intervention designed to enhance romantic relationship excitement: A randomized-control trial. *Couple Fam. Psychol.: Res. Pract*. **2**, 34–44; <https://doi.org/10.1037/a0031719> (2013).
111. Rosa, M. N. *et al*. Encouraging erotic variety: Identifying correlates of, and strategies for promoting, sexual novelty in romantic relationships. *Pers. Individ. Diff*. **146**, 158-169; <https://doi.org/10.1016/j.paid.2019.04.009> (2019).
112. Emmers, T. A. & Dindia, K. The effect of relational stage and intimacy on touch: An extension of Guerrero and Andersen. *Pers. Relatsh*. **2**, 225-236; <https://doi.org/10.1111/j.1475-6811.1995.tb00088.x> (1995).
113. Heiman, J. R., Long, J. S., Fisher, W. A., Sand, M. S. & Rosen, R. C. Sexual satisfaction and relationship happiness in midlife and older couples in five countries. *Arch. Sex. Behav*. **40**, 741-753; <https://doi.org/10.1007/s10508-010-9703-3> (2011).
114. Leavitt, C. E. & Willoughby, B. J. Associations between attempts at physical intimacy and relational outcomes among cohabiting and married couples. *J. Soc. Pers. Relatsh*. **32**, 241-262; <https://doi.org/10.1177/0265407514529067> (2015).
115. Sorokowska, A. *et al*. Love and affectionate touch toward romantic partners all over the world. *Sci. Rep*. **13**, 5497; <https://doi.org/10.1038/s41598-023-31502-1> (2023).
116. Gagné, F. M. & Lydon, J. E. Identification and the commitment shift: Accounting for gender differences in relationship illusions. *Pers. Soc. Psychol. Bull*. **29**, 907-919; <https://doi.org/10.1177/0146167203029007009> (2003).
117. Murray, S. L., Holmes, J. G. & Griffin, D. W. The benefits of positive illusions: Idealization and the construction of satisfaction in close relationships. *J. Pers. Soc. Psychol*. **70**, 79-98; <https://doi.org/10.1037/0022-3514.70.1.79> (1996).
118. Murray, S. L., Holmes, J. G. & Griffin, D. W. The self-fulfilling nature of positive illusions in romantic relationships: Love is not blind, but prescient. J. Pers. Soc. Psychol. **71**, 1155-1180; <https://doi.org/10.1037/0022-3514.71.6.1155> (1996).
119. Niehuis, S., Lee, K.-H., Reifman, A., Swenson, A. & Hunsaker, S. Idealization and disillusionment in intimate relationships: A review of theory, method, and research. *J. Fam. Theory Rev*. **3**, 273-302; <https://doi.org/10.1111/j.1756-2589.2011.00100.x> (2011).
120. Star, A. P., Cohn-Schwartz, E. & O’Rourke, N. Reciprocal effects of marital idealization and marital satisfaction between long-wed spouses over time. *Int. J. Aging Hum. Dev*. **95**, 440-454; <https://doi.org/10.1177/00914150221077953> (2022).
121. Buss, D. M. & Schmitt, D. P. Sexual strategies theory: an evolutionary perspective on human mating. *Psychol. Rev*. **100**, 204-232; <https://doi.org/10.1037/0033-295x.100.2.204> (1993).
122. Durkee, P. K. *et al*. Men’s bodily attractiveness: Muscles as fitness indicators. *Evol. Psychol*. **17**, <https://doi.org/10.1177/1474704919852918> (2019).
123. Lewis, D. M. G. *et al*. Friends with benefits: The evolved psychology of same- and opposite-sex friendship. *Evol. Psychol*. **9**, 543-563; <https://doi.org/10.1177/147470491100900407> (2011).
124. Perilloux, C. & Buss D. M. Breaking up romantic relationships: Costs experienced and coping strategies deployed. *Evol. Psychol*. **6**, <https://doi.org/10.1177/147470490800600119> (2008).
125. Conradi, H. J., Noordhof, A., Boyette, L.-L. & de Jonge, P. Physical distance between romantic partners as a marker for attachment in couples: A proof of concept study. *Aust. N. Z. J. Fam. Ther.* **41**, 91-106; <https://doi.org/10.1002/anzf.1398> (2020).
126. Epstein, R. How science can help you fall in love. *Scientific American Mind* <https://drrobertepstein.com/downloads/Epstein-HOW_SCIENCE_CAN_HELP_YOU_FALL_IN_LOVE-Sci_Am_Mind-JanFeb2010.pdf> (2010).
127. Festinger, L., Schachter, S. & Back, K. W. *Social pressures in informal groups: A study of human factors in housing* (Harper & Brothers, 1950).
128. Gilbertson, J., Dindia, K. & Allen, M. Relational continuity constructional units and the maintenance of relationships. *J. Soc. Pers. Relatsh*. **15**, 774-790; <https://doi.org/10.1177/0265407598156004> (1998).
129. Hall, E. T. *The hidden dimension* (Doubleday, 1966).
130. Buss, D. M. & Schmitt, D. P. Mate preferences and their behavioral manifestations. *Annu. Rev. Psychol*. **70**, 77-110; <https://doi.org/10.1146/annurev-psych-010418-103408> (2019).
131. Gottman, J. M. & Levenson, R. W. Marital processes predictive of later dissolution: Behavior, physiology, and health. *J. Pers. Soc. Psychol*. **63**, 221–233; <https://doi.org/10.1037/0022-3514.63.2.221> (1992).
132. Hitsch, G. J., Hortacsu, A. & Ariely, D. What makes you click? Mate preferences and matching outcomes in online dating. Preprint at <https://dx.doi.org/10.2139/ssrn.895442> (2006).
133. Mathes, E. W. & Kozak, G. The exchange of physical attractiveness for resource potential and commitment. *J. Evol. Psychol*. **6**, 43-56; <https://doi.org/10.1556/jep.2008.1004> (2008).
134. Impett, E. A., Gable, S. L. & Peplau, L. A. Giving up and giving in: the costs and benefits of daily sacrifice in intimate relationships. *J. Pers. Soc. Psychol*. **89**, 327-344; <https://doi.org/10.1037/0022-3514.89.3.327> (2005).
135. Righetti, F. & Impett, E. Sacrifice in close relationships: Motives, emotions, and relationship outcomes. *Soc. Pers. Psychol. Com*. **11**, e12342; <https://doi.org/10.1111/spc3.12342> (2017).
136. Zoppolat, G., Visserman, M. L. & Righetti, F. A nice surprise: Sacrifice expectations and partner appreciation in romantic relationships. *J. Soc. Pers. Relatsh*. **37**, 450-466; <https://doi.org/10.1177/0265407519867145> (2019).
137. Righetti, F., Visserman, M. L. & Impett, E. A. Sacrifices: Costly prosocial behaviors in romantic relationships. *Curr. Opin. Psychol*. **44**, 74-79; <https://doi.org/10.1016/j.copsyc.2021.08.031> (2022).
138. Aron, A., Melinat, E., Aron, E. N., Vallone, R. D. & Bator, R. J. The experimental generation of interpersonal closeness: A procedure and some preliminary findings. *Pers. Soc. Psychol. Bull*. **23**, 363-377; <https://doi.org/10.1177/0146167297234003> (1997).
139. Brummelman, E., Bos, P. A., de Boer, E., Nevicka, B. & Sedikides, C. Reciprocal self-disclosure makes children feel more loved by their parents in the moment: A proof-of-concept experiment. *Dev. Sci*. **27**, e13516; <https://doi.org/10.1111/desc.13516> (2024).
140. Chen, Q., Zhang, Q., Zhao, S. & Li, C. Trusting strangers: The benefits of reciprocal self-disclosure during online computer-mediated communication and mediating role of interpersonal liking. *Int. J. Psychol*. **59**, 143-154; <https://doi.org/10.1002/ijop.12957> (2023).
141. Collins, N. L. & Miller, L. C. Self-disclosure and liking: A meta-analytic review. *Psychol. Bull*. **116**, 457-475; <https://doi.org/10.1037/0033-2909.116.3.457> (1994).
142. Finkenauer, C. & Hazam, H. Disclosure and secrecy in marriage: Do both contribute to marital satisfaction? *J. Soc. Pers. Relatsh*. **17**, 245-263; <https://doi.org/10.1177/0265407500172005> (2000).
143. Khalifian, C. E. & Barry, R. A. Expanding intimacy theory: Vulnerable disclosures and partner responding. *J. Soc. Pers. Relatsh*. **37**, 58-76; <https://doi.org/10.1177/0265407519853047> (2019).
144. Keelan, J. P. R., Dion, K. K. & Dion, K. L. Attachment style and relationship satisfaction: Test of a self-disclosure explanation. *Can. J. Behav. Sci*. **30**, 24–35; <https://psycnet.apa.org/doi/10.1037/h0087055> (1998).
145. Sprecher, S. & Hendrick, S. S. Self-disclosure in intimate relationships: Associations with individual and relationship characteristics over time. *J. Soc. Clin. Psychol*. **23**, 857-877; <https://doi.org/10.1521/jscp.23.6.857.54803> (2004).
146. Sprecher, S., Treger S. & Wondra, J. D. Effects of self-disclosure role on liking, closeness, and other impressions in get-acquainted interactions. *J. Soc. Pers. Relatsh*. **30**, 497-514; <https://doi.org/10.1177/0265407512459033> (2012).
147. Welker, K. M. *et al.* Effects of self-disclosure and responsiveness between couples on passionate love within couples. *Pers. Relatsh*. **21**, 692-708; <https://doi.org/10.1111/pere.12058> (2014).
148. Canary, D. J., Stafford, L. & Semic, B. A. A panel study of the associations between maintenance strategies and relational characteristics. *J. Marriage Fam*. **64**, 395-406; <https://doi.org/10.1111/j.1741-3737.2002.00395.x> (2004).
149. Oswald, D. L., Clark, E. M. & Kelly, C. M. Friendship maintenance: An analysis of individual and dyad behaviors. *J. Soc. Clin. Psychol*. **23**, 413-441; <https://doi.org/10.1521/jscp.23.3.413.35460> (2005).
150. Park, C., Harris, V. W. & Duncan, J. C. The association between mindfulness and couple quality: The mediating roles of self-care and engagement in shared relationship activities. *J. Fam. Ther*. **46**, 216-230. <https://doi.org/10.1111/1467-6427.12462> (2024).
151. Weigel, D. J. A dyadic assessment of how couples indicate their commitment to each other. *Pers. Relatsh*. **15**, 17-39; <https://doi.org/10.1111/j.1475-6811.2007.00182.x> (2008).
152. Wolf, W., Launay, J. & Dunbar, R. I. M. Joint attention, shared goals, and social bonding. *Br. J. Psychol*. **107**, 322-337; <https://doi.org/10.1111/bjop.12144> (2015).
153. Blair, K. L. & Holmberg, D. Perceived social network support and well-being in same-sex versus mixed-sex romantic relationships. *J. Soc. Pers. Relatsh*. **25**, 769-791; <https://doi.org/10.1177/0265407508096695> (2008).
154. Blair, K. L., Hudson, C. & Holmberg, D. Walking hand in hand: The role of affection-sharing in understanding the social network effect in same-sex, mixed-sex, and gender-diverse relationships. *J. Soc. Pers. Relatsh*. **40**, 3171-3194; <https://doi.org/10.1177/02654075231169786> (2023).
155. Felmlee, D. H. No couple is an island: A social network perspective on dyadic stability. *Soc. Forces*. **79**, 1259-1287; <https://doi.org/10.1353/sof.2001.0039> (2001).
156. Shulman, S. *et al.* Adolescent romantic competence and parenting attitudes: Gender variations and correlates. *J. Soc. Pers. Relatsh*. **34**, 594-614; <https://doi.org/10.1177/0265407516650760> (2016).
157. Sprecher, S. & Felmlee, D. The influence of parents and friends on the quality and stability of romantic relationships: A three-wave longitudinal investigation. *J. Marriage Fam*. **54**, 888-900; <https://doi.org/10.2307/353170> (1992).
158. Cramer, D. & Jowett, S. Perceived empathy, accurate empathy and relationship satisfaction in heterosexual couples. *J. Soc. Pers. Relatsh*. **27**, 327-349; <https://doi.org/10.1177/0265407509348384> (2010).
159. Grueneisen, S. & Warneken, F. (2022). The development of prosocial behavior—from sympathy to strategy. *Curr. Opin. Psychol*. **43**, 323-328; <https://doi.org/10.1016/j.copsyc.2021.08.005>
160. Poucher, J., Prager, K. J., Shirvani, F., Parsons, J. & Patel, J. Intimacy, attachment to the partner, and daily well-being in romantic relationships. *J. Soc. Pers. Relatsh*. **39**, 1574-1601; <https://doi.org/10.1177/02654075211060392> (2022).
161. Schmidt, C. D. & Gelhert, N. C. Couples therapy and empathy. *The Family Journal*. **25**, 23-30; <https://doi.org/10.1177/1066480716678621> (2016).
162. Ulloa, E. C., Hammett, J. F., Meda, N. A. & Rubalcaba, S. J. Empathy and romantic relationship quality among cohabitating couples: An actor–partner interdependence model. *The Family Journal*. **25**, 208-214; <https://doi.org/10.1177/1066480717710644> (2017).
163. Algoe, S. B., Gable, S. L. & Maisel, N. C. It's the little things: Everyday gratitude as a booster shot for romantic relationships. *Pers. Relatsh*. **17**, 217-233; <https://doi.org/10.1111/j.1475-6811.2010.01273.x> (2010).
164. Ogolsky, B. G., Monk, J. K., Rice, T. M., Theisen, J. C. & Maniotes, C. R. Relationship maintenance: A review of research on romantic relationships. *J. Fam. Theory Rev*. **9**, 275-306; <https://doi.org/10.1111/jftr.12205> (2017).
165. Büchi, S. *et al*. Shared or discordant grief in couples 2–6 years after the death of their premature baby: Effects on suffering and posttraumatic growth. *Psychosomatics*. **50**, 123-130; <https://doi.org/10.1176/appi.psy.50.2.123> (2009).
166. Elder, G. H. Jr. & Clipp, E. C. Wartime losses and social bonding: Influences across 40 years in men's lives. *Psychiatry*. **51**, 177-198; <https://doi.org/10.1080/00332747.1988.11024391> (1988).
167. White, C. *et al*. How shared suffering bonded Britons witnessing the Queen’s funeral. *Sci. Rep*. **14**, 16620; <https://doi.org/10.1038/s41598-024-66537-5> (2024).
168. Williamson, H. C., Bradbury, T. N. & Karney, B. R. Experiencing a natural disaster temporarily boosts relationship satisfaction in newlywed couples. *Psychol. Sci*. **32**, 1709-1719; <https://doi.org/10.1177/09567976211015677> (2021).
169. Coffey, J. K., Shahvali, M., Kerstetter, D. & Aron, A. Couples vacations and romantic passion and intimacy. *Ann. Tour. Res. Empir. Insights*. **5**, 100121; <https://doi.org/10.1016/j.annale.2024.100121> (2024).
170. Shahvali, M., Kerstetter, D. L. & Townsend, J. N. The contribution of vacationing together to couple functioning. *J. Travel Res*. **60**, 113-148; <https://doi.org/10.1177/0047287519892340> (2019).
171. Baumeister, R. F. & Leary, M. R. The need to belong: Desire for interpersonal attachments as a fundamental human motivation. *Psychol. Bull*. **117**, 497-529; <https://doi.org/10.1037/0033-2909.117.3.497> (1995).
172. Hazan, C. & Campa, M. I. (eds.) *Human bonding: The science of affectional ties*. (The Guilford Press, 2013).
173. Sterck, E. H. M. *et al*. The evolution of between-sex bonds in primates. *Evol. Hum. Behav.* **45**, <https://doi.org/10.1016/j.evolhumbehav.2024.106628> (2024).
174. Carter, C. S. Oxytocin and love: Myths, metaphors and mysteries. *Compr. Psychoneuroendocrinology*. **9**, <https://doi.org/10.1016/j.cpnec.2021.100107> (2022).
175. Birnbaum, G. E. & Reis, H. T. When does responsiveness pique sexual interest? Attachment and sexual desire in initial acquaintanceships. *Pers. Soc. Psychol. Bull*. **38**, 946-958; <https://doi.org/10.1177/0146167212441028> (2012).
176. Muise, A., Impett, E. A. & Desmarais, S. Getting it on versus getting it over with: Sexual motivation, desire, and satisfaction in intimate bonds. *Pers. Soc. Psychol. Bull*. **39**, 1320-1332; <https://doi.org/10.1177/0146167213490963> (2013).
177. Layman, M. J., Gidycz, C. A. & Lynn, S. J. Unacknowledged versus acknowledged rape victims: Situational factors and posttraumatic stress. *J. Abnorm. Psychol*. **105**, 124-131; <https://doi.org/10.1037/0021-843X.105.1.124> (1996).
178. Hennig, C. W. Effects of simulated predation on tonic immobility in Anolis carolinensis: The role of eye contact. *Bull. Psychon. Soc*. **9**, 239-242; <https://doi.org/10.3758/BF03336987> (1977).
179. Nahm, F. K. D., Perret, A., Amaral, D. G. & Albright, T. D. How do monkeys look at faces? *J. Cogn. Neurosci*. **9**, 611-623; <https://doi.org/10.1162/jocn.1997.9.5.611> (1997).
180. Skuse, D. Fear recognition and the neural basis of social cognition. *Child and Adolesc. Ment. Health*. **8**, 50-60; <https://doi.org/10.1111/1475-3588.00047> (2003).
181. Whitham, W. *et al*. Predator gaze captures both human and chimpanzee attention. *PLOS ONE*. **19**, e0311673; <https://doi.org/10.1371/journal.pone.0311673> (2024).
182. Ley, A. Does anyone know how to behave on the subway anymore? *The New York Times*. <https://www.nytimes.com/2023/11/07/nyregion/subway-nyc-rules-conduct.html> (2023).
183. Ewing, L., Rhodes, G. & Pellicano, E. Have you got the look? Gaze direction affects judgements of facial attractiveness. *Vis. Cogn*. **18**, 321-330. <https://doi.org/10.1080/13506280902965599> (2009).
184. Kreysa, H., Kessler, L. & Schweinberger, S. R. Direct speaker gaze promotes trust in truth-ambiguous statements. *PLOS ONE*. **11**, e0162291; <https://doi.org/10.1371/journal.pone.0162291> (2016).
185. Castleman, M. Why gazing into a partner’s eyes boosts intimacy and sexual pleasure. *Psychology Today*. <https://www.psychologytoday.com/us/blog/all-about-sex/202108/why-gazing-partner-s-eyes-boosts-intimacy-and-sexual-pleasure> (2021).
186. Brown, B. The power of vulnerability. *TED Conferences*. <https://www.ted.com/talks/brene_brown_the_power_of_vulnerability> (2010).
187. Brown, B. *Daring greatly: How the courage to be vulnerable transforms the way we live, love, parent and lead*. (Penguin Random House, 2012).
188. Louisiana Contextual Science Research Group Promoting appetitive learning of consensual, empowered vulnerability: A contextual behavioral conceptualization of intimacy. *Front. Psychol*. **14**, <https://doi.org/10.3389/fpsyg.2023.1200452> (2023).
189. Kardan-Souraki, M., Hamzehgardeshi, Z., Asadpour, I., Mohammadpour, R. A. & Khani, S. A review of marital intimacy-enhancing interventions among married individuals. *Glob. J. Health Sci*. **8**, 74-93; <https://doi.org/10.5539/gjhs.v8n8p74> (2016).
190. Bodenmann G., Kessler, M., Kuhn, M., Hocker, L. & Randall, A. K. Cognitive-behavioral and emotion-focused couple therapy: Similarities and differences. *Clin. Psychol. Eur*. **2**, e2741; <https://doi.org/10.32872/cpe.v2i3.2741> (2020).
191. McKinnon, M. K. & Greenberg, L. S. Vulnerable emotional expression in emotion focused couples therapy: Relating interactional processes to outcome. *J. Marital Fam. Ther*. **43**, 198-212; <https://doi.org/10.1111/jmft.12229> (2017).
192. Baghramian, M., Petherbridge, D. & Stout, R. Vulnerability and trust: An introduction. *Int. J. Philos. Stud*. **28**, 575-582; <https://doi.org/10.1080/09672559.2020.1855814> (2020).
193. Bruk, A., Scholl, S. G. & Bless, H. You and I both: Self-compassion reduces self–other differences in evaluation of showing vulnerability. *Pers. Soc. Psychol. Bull*. **48**, 1054-1067; <https://doi.org/10.1177/01461672211031080> (2021).
194. Yamaguchi, S., Kawata, Y., Murofushi, Y., Shibata, N. & Ota, T. Psychological vulnerability associated with stress coping strategies in Japanese university athletes. *J. Clin. Sport Psychol.* **14**, 449-463; <https://doi.org/10.1123/jcsp.2021-0084> (2022).
195. Younie, L. Vulnerable leadership. *LJPC*. **8**, 37-38; <https://doi.org/10.1080/17571472.2016.1163939> (2016).
